# Structural diversity in copper(I) iodide complexes with 6-thioxopiperidin-2-one, piperidine-2,6-di­thione and isoindoline-1,3-di­thione ligands

**DOI:** 10.1107/S2056989020009676

**Published:** 2020-07-21

**Authors:** Amelia M. Wheaton, Ilia A. Guzei, John F. Berry

**Affiliations:** a University of Wisconsin-Madison, Department of Chemistry, 1101 University Avenue, Madison, WI, 53703, USA

**Keywords:** crystal structure, copper(I) iodide, coordination complexes

## Abstract

Copper(I) iodide complexes are well known for displaying a diverse array of structural features even when only small changes in ligand design are made. This structural diversity is well displayed by five copper(I) iodide compounds reported here with closely related piperidine-2,6-di­thione (SNS), isoindoline-1,3-di­thione (SNS6), and 6-thioxopiperidin-2-one (SNO) ligands: di-μ-iodido-bis­[(aceto­nitrile-κ*N*)(6-sulfanylidenepiperidin-2-one-κ*S*)copper(I)], [Cu_2_I_2_(CH_3_CN)_2_(C_5_H_7_NOS)_2_] (**I**), bis­(aceto­nitrile-κ*N*)tetra-μ_3_-iodido-bis­(6-sulfanylidenepiperidin-2-one-κ*S*)-*tetra­hedro*-tetra­copper(I), [Cu_4_I_4_(CH_3_CN)_4_(C_5_H_7_NOS)_4_] (**II**), *catena*-poly[[(μ-6-sulfanylidenepiperidin-2-one-κ^2^ *O*:*S*)copper(I)]-μ_3_-iodido], [CuI(C_5_H_7_NOS)]_*n*_ (**III**), poly[[(piperidine-2,6-di­thione-κ*S*)copper(I)]-μ_3_-iodido], [CuI(C_5_H_7_NS_2_)]_*n*_ (**IV**), and poly[[(μ-isoindoline-1,3-di­thione-κ^2^ *S*:*S*)copper(I)]-μ_3_-iodido], [CuI(C_8_H_5_NS_2_)]_*n*_ (**V**). Compounds **I** and **II** crystallize as discrete dimeric and tetra­meric complexes, whereas **III**, **IV**, and **V** crystallize as polymeric two-dimensional sheets. To the best of our knowledge, compound **III** is the first instance of an extended hexa­gonal [Cu_3_I_3_] structure that is not supported by bridging ligands. Structures **I**, **II**, and **IV** display weak to moderately strong Cu⋯Cu cuprophilic inter­actions [Cu⋯Cu inter­nuclear distances range between 2.5803 (10) and 2.8485 (14) Å]. All structures except **III** display weak hydrogen-bonding inter­actions between the N—H of the ligand and the μ_2_ and μ_3_-I^−^ atoms. Structure **III** contains classical N–H⋯O inter­actions between the SNO ligands that connect the mol­ecules in a three-dimensional framework. Complex **V** features π–π stacking inter­actions between the aryl rings of the SNS6 ligands within the same polymeric sheet. In structure **IV**, there were three partially occupied solvent mol­ecules of di­chloro­methane and one partially occupied mol­ecule of aceto­nitrile present in the asymmetric unit. The SQUEEZE routine [Spek (2015 ▸). *Acta Cryst*. C**71**, 9–18] was used to correct the diffraction data for diffuse scattering effects and to identify the solvent mol­ecules. The given chemical formula and other crystal data do not take into account the solvent mol­ecules.

## Abstract 2

Five copper(I) iodide coordination compounds were synthesized and characterized by single-crystal X-ray diffraction measurements; the resulting structures display a diverse array of structural features.

## Chemical context   

**Figure 1:**
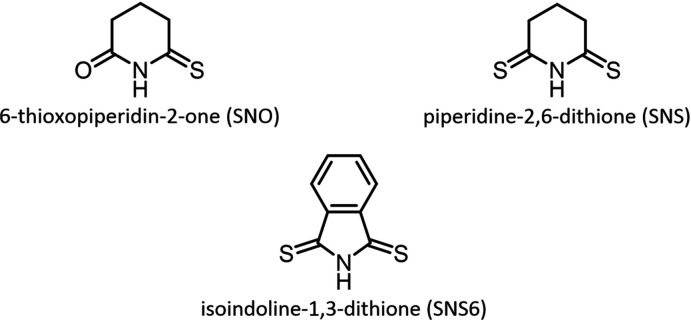
Diagrams of the three ligands used in the preparation of structures **I**–**V**.

Copper (I) iodide compounds have been of inter­est for the past 50 years because of their diverse structural (Peng *et al.*, 2010 ▸) and spectroscopic properties (Ford *et al.*, 1999 ▸; Hardt & Pierre, 1973 ▸). In particular, Cu^I^ complexes range from simple Cu_2_I_2_ *L*_2_ dimers (*L* = Lewis basic ligands) to complex three-dimensional coordination polymers (Peng *et al.*, 2010 ▸). Traditionally, soft Lewis basic donors such as thiols or phosphines have been used as ligands to the Cu^I^ centers. We were inter­ested in exploring the structures of Cu^I^ coordination complexes with three ligands, piperidine-2,6-di­thione (SNS), isoindoline-1,3-di­thione (SNS6), and 6-thioxopiperidin-2-one (SNO) (Fig. 1 ▸). These ligands have been previously utilized in our work due to their polydentate binding modes, which provide individual binding sites that display a range of ‘hard’ to ‘soft’ Lewis basic behavior (Dolinar & Berry, 2013 ▸, 2014 ▸). Herein we report the synthesis and structural characterization of a series of five copper(I) iodide complexes with piperidine-2,6-di­thione (SNS), isoindoline-1,3-di­thione (SNS6), and 6-thioxopiperidin-2-one (SNO) ligands.
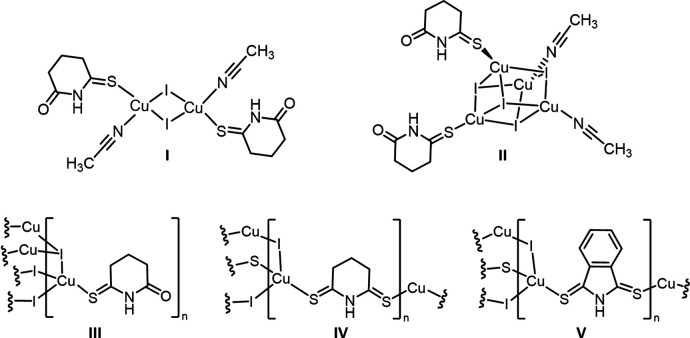


## Structural commentary   

**Figure 2:**
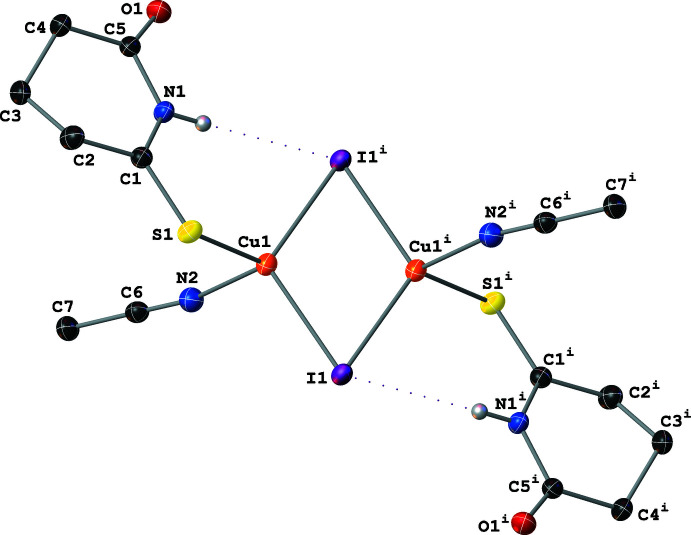
A mol­ecular drawing of **I** with 50% probability ellipsoids. Dotted lines are used to indicate hydrogen-bonding inter­actions. All H atoms bound to C atoms are omitted. [Symmetry code: (i) −*x* + 1, −*y* + 1, −*z*.]

Compound **I** crystallizes as a discrete dimer with a rhombic Cu_2_(μ_2_-I)_2_ core that resides on a crystallographic inversion center; thus, only one half of the dimer is symmetry-independent (Fig. 2 ▸). The rhombic core is close to having an ideal geometry with almost equal Cu—I distances (Table 1 ▸). Each Cu center is coordinated by two μ_2_-I^−^ atoms, one mol­ecule of aceto­nitrile, and the thione moiety of the SNO ligand and has a slightly distorted tetra­hedral geometry (I—Cu—I and I—Cu—*L* angles of 100.19 (3)–118.719 (16)°; *L* = SNO or aceto­nitrile). The Cu⋯Cu inter­nuclear distance of 2.7274 (6) Å is slightly shorter than the sum of the covalent radii (*ca* 2.87 Å) and is consistent with a weak cuprophilic inter­action*.* The Cu—N and Cu—S distances (Table 1 ▸) in **I** are similar to the Cu—N and Cu—S distances in other discrete Cu_2_(μ_2_-I)_2_ dimers reported to the Cambridge Structural Database (CSD) and selected with moderate search criteria (Groom *et al.* 2016 ▸; no errors, no polymers, single-crystal structures only). The SNO ligand adopts an envelope conformation, with a 49.07 (9)° dihedral angle between the planes defined by atoms C2–C3–C4 and C2–C1–N1–C5–C4.

**Figure 3:**
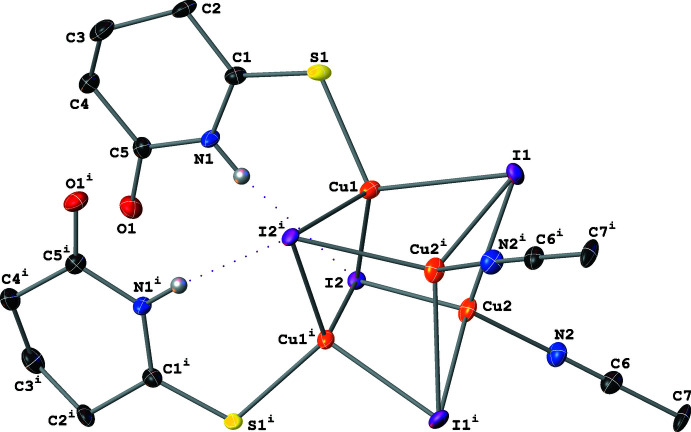
A mol­ecular drawing of **II** shown with 50% probability ellipsoids. Dotted lines are used to indicate hydrogen-bonding inter­actions. All H atoms bound to C atoms are omitted. [Symmetry code: (i) −*x* + 1, *y*, −*z* + 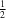.]

Complex **II** crystallizes with a Cu_4_(μ_3_-I)_4_ core; the four Cu atoms form a distorted tetra­hedron with μ_3_-I^−^ atoms capping each of the tetra­hedron faces (Fig. 3 ▸). The center of the tetra­hedron resides on a crystallographic twofold axis and therefore only two of the Cu centers are symmetry-independent. These two Cu atoms have different first coordination spheres: Cu1 is coordinated by three μ_3_-I^−^ atoms and one thione-bound SNO ligand; Cu2 is coordinated by three μ_3_-I^−^ atoms and one aceto­nitrile ligand. Both Cu atoms have a distorted tetra­hedral geometry [I—Cu—I and I—Cu—*L* angles between 97.98 (3) and 118.71 (2)°; *L* = SNO or aceto­nitrile]. The inter­nuclear Cu⋯Cu distances vary between 2.5803 (10) and 2.8150 (11) Å (Table 1 ▸), which (similarly to **I**) are indicative of weak to moderately strong cuprophilic inter­actions between Cu atoms in the tetra­hedron. The Cu1—S and Cu2—N distances in **II** (Table 1 ▸) are slightly shorter than the Cu—S and Cu—N distances in **I** as a result of the increase from two μ_2_-I^−^ to three μ_3_-I^−^ atoms coordinating to each Cu center. The SNO ligand adopts an envelope conformation with a 47.5 (2)° dihedral angle between the planes defined by atoms C2–C3–C4 and C2–C1–N1–C5–C4.

**Figure 4:**
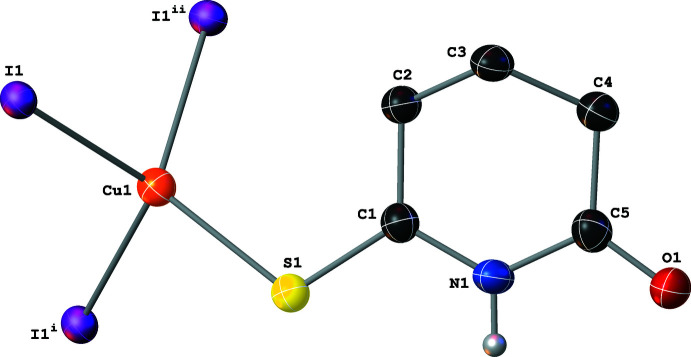
A mol­ecular drawing of the symmetry-independent portion of **III** with the full coordination sphere of the Cu center shown. All atoms are shown with 50% probability ellipsoids; all H atoms bound to C atoms are omitted. [Symmetry codes: (i) *x*, −*y* + 1, *z* − 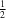; (ii) −*x* + 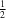, −*y* + 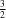, *z* − 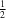.]

**Figure 5:**
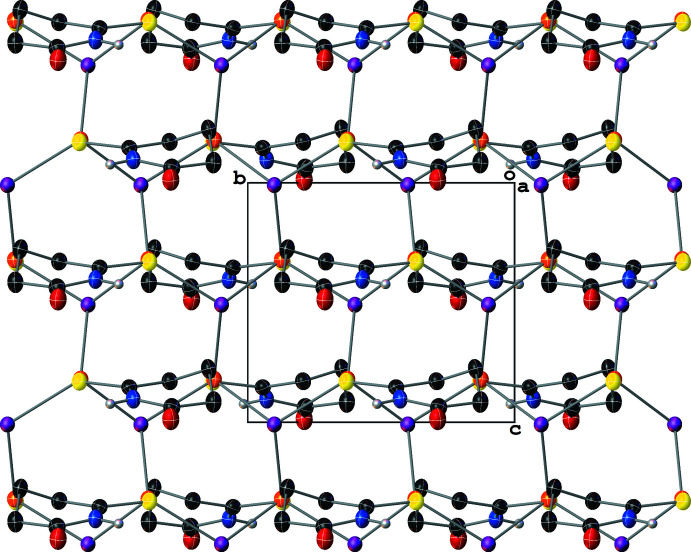
A mol­ecular drawing of **III**’s Cu_3_I_3_ fused rings viewed along the crystallographic *a* axis with 50% probability ellipsoids. All H atoms bound to C atoms are omitted.

**Figure 6:**
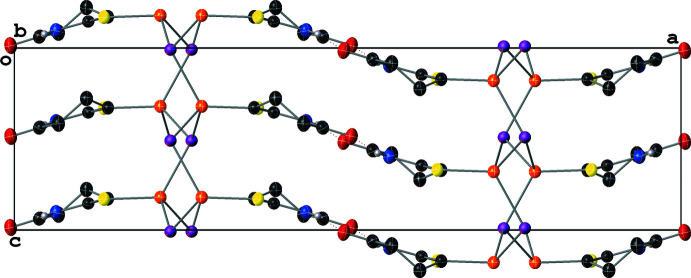
A mol­ecular drawing of **III** viewed along the crystallographic *b* axis with 50% probability ellipsoids. Dotted lines are used to indicate hydrogen-bonding inter­actions. All H atoms bound to C atoms are omitted.

Compound **III** crystallizes with layered two-dimensional polymeric sheets with a repeat (and symmetry-independent) unit formula of [Cu(μ_3_-I)(SNO)]. The Cu atoms are coordinated by three μ_3_-I^−^ atoms and one SNO ligand and have distorted tetra­hedral geometries [I—Cu—I and I—Cu—S angles of 97.12 (4)–120.62 (4)°] (Fig. 4 ▸). The I^−^ ions have distorted trigonal pyramidal geometries [Cu—I—Cu angles of 99.58 (3)–116.92 (2)°] with two short and one long Cu—I bonds (Table 1 ▸). The polymeric sheet is based on fused Cu_3_I_3_ six-membered rings with a screw-boat conformation (^3^ *S*_2_ with puckering amplitude *Q* = 1.3385 Å; Cremer & Pople, 1975 ▸) that propagate parallel to and stack perpendicularly to the (100) crystallographic plane (Fig. 5 ▸). These fused six-membered rings are reminiscent of the zinc-blend structure present in crystalline γ-CuI (Gruzintsev & Zagorodnev, 2012 ▸) except that the anions are μ_3_ rather than μ_4_. Each polymeric sheet is insulated by a sheath of SNO ligands, whose Cu—S bonds are perpendicular to the plane of propagation of the Cu_3_I_3_ rings (Fig. 6 ▸). The Cu⋯Cu distances between neighboring Cu atoms in the Cu_3_I_3_ rings measure between 4.2226 (15) and 4.5148 (15) Å, which are outside the range of inter­nuclear distances for cuprophilic inter­actions.

**Figure 7:**
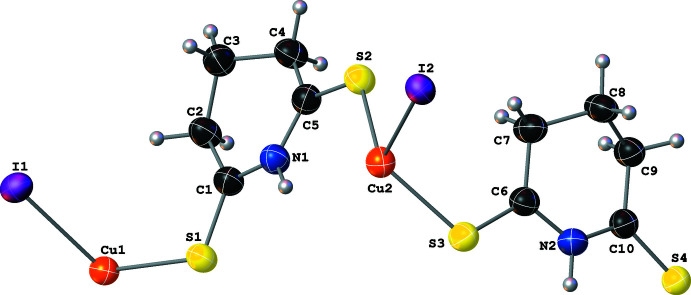
A mol­ecular drawing of the repeat unit of **IV** shown with 50% probability ellipsoids.

**Figure 8:**
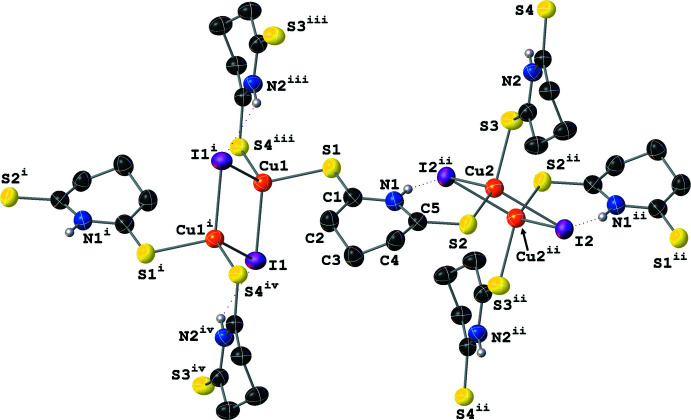
A mol­ecular drawing of **IV** with the full coordination spheres of the Cu centers shown with selected atom labels. All atoms are shown with 50% probability ellipsoids; dotted lines are used to indicate hydrogen-bonding inter­actions. All H atoms bound to C atoms are omitted. [Symmetry codes: (i) −*x*, −*y*, 1 − *z*; (ii) 1 − *x*, 1 − *y*, 1 − *z*; (iii) 1 − *x*, 1 − *y*, 1 − *z*; (iv) −1 + *x*, *y*, *z*]

Similarly to **III**, **IV** crystallizes with layered two-dimensional polymeric sheets with the symmetry-independent unit formula [Cu(μ_2_-I)(μ_2_-SNS)]_2_ (Fig. 7 ▸); the Cu and μ_2_-I^−^ atoms form Cu_2_(μ_2_-I)_2_ rhombi where the center of each rhombus resides on a crystallographic inversion center. Thus, the symmetry-independent unit is best described as containing two structurally distinct [Cu_2_(μ_2_-I)_2_(μ_2_-SNS)_2_] half-dimers. The structures of the symmetry-independent Cu_2_(μ_2_-I)_2_ rhombi differ in two notable ways: first, while the Cu_2_(μ_2_-I)_2_ rhombus formed by Cu1, I1, and their symmetry-equivalents is slightly distorted, the rhombus formed by Cu2, I2, and their symmetry-equivalents is near ideal (Table 1 ▸). Secondly, the Cu⋯Cu distances in the rhombi differ by *ca* 0.07 Å [2.8485 (14) Å for the Cu1 rhombus; 2.7746 (15) Å for the Cu2 rhombus]. These values are consistent with little to no Cu⋯Cu cuprophilic inter­action in the Cu1 dimer while also indicating that there is a weak Cu⋯Cu cuprophilic inter­action in the Cu2 dimer. For both half dimers, the Cu atom’s distorted tetra­hedral [I—Cu—I angles between 115.06 (3) and 117.46 (3)° and S—Cu—I angles of 96.95 (4)–119.35 (6)°] coordination sphere is filled by two thione moieties from the μ_2_-SNS ligand; however, only one of these μ_2_-SNS ligands per Cu atom is symmetry-independent (Fig. 8 ▸).

**Figure 9:**
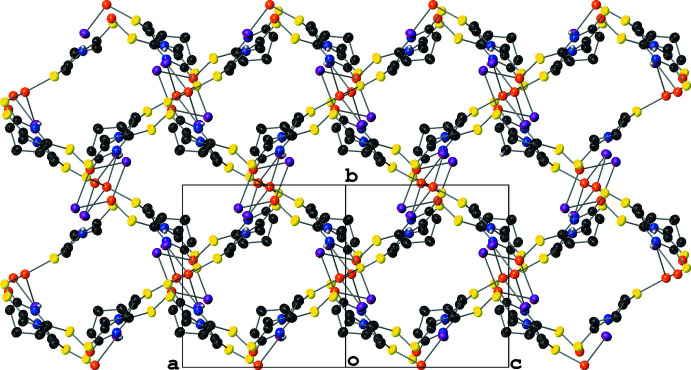
A mol­ecular drawing of **IV** viewed along the [101] crystallographic direction with 50% probability ellipsoids. All H atoms bound to C atoms are omitted.

In contrast to the monodentate SNO ligands in **III**, which only permit polymer propagation in **III** through the μ_3_-I^−^ atoms, the bidentate SNS ligand facilitates polymer propagation in **IV**. This results in the formation of rings formed by four [Cu(μ_2_-I)_2_(μ_2_-SNS)] units. The propagation of these rings in the (001) crystallographic plane results in a mesh-like sheet structure, and the layering of these sheets perpendicularly to the (001) plane results in the presence of sizable solvent-accessible voids (*ca* 200 Å^3^) in the structure (Fig. 9 ▸). These voids are filled with a combination of aceto­nitrile and di­chloro­methane in an approximately 2:1 ratio; however, these solvent species were positionally disordered and the *PLATON* SQUEEZE routine (Spek, 2015 ▸) was required to model the diffuse electron density from the solvent species in these voids (see *Refinement* section).

**Figure 10:**
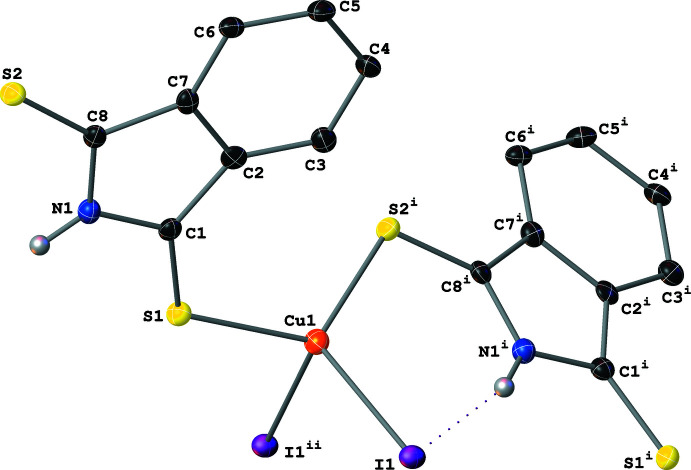
A mol­ecular drawing of the symmetry-independent portion of **V** with the full coordination sphere of the Cu center shown. All atoms are shown with 50% probability ellipsoids; dotted lines are used to indicate hydrogen-bonding inter­actions. All H atoms bound to C atoms are omitted. [Symmetry codes: (i) *x*, 1 + *y*, *z*; (ii) *x*, 1 − *y*, 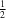 + *z.*]

Complex **V** also crystallizes as two-dimensional polymeric sheets with the symmetry-independent unit formula [Cu(μ_2_-I)(μ_2_-SNS6)] (Fig. 10 ▸). The Cu center is coordinated by two μ_2_-I^−^ atoms and two thione moieties of the μ_2_-SNS6 ligands and has a distorted tetra­hedral geometry [I—Cu—I and I—Cu—S angles between 100.30 (6) and 120.16 (7)°]. Whereas the two S—Cu distances are almost identical, the two Cu—I distances are quite different (Table 1 ▸).

**Figure 11:**
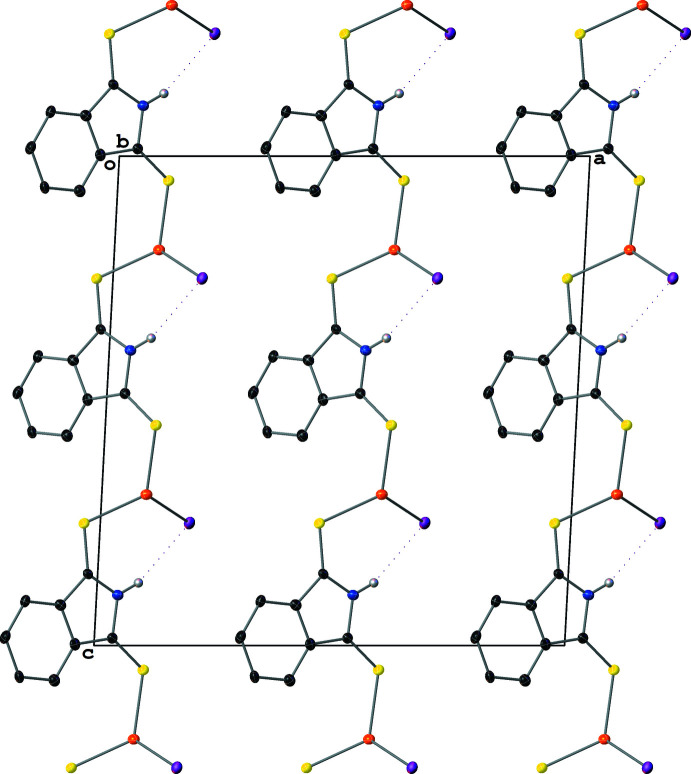
A mol­ecular drawing of **V** viewed along the crystallographic *b* axis with 50% probability ellipsoids with emphasis on the weak N—H⋯I inter­actions (dotted lines). All H atoms bound to C atoms are omitted.

**Figure 12:**
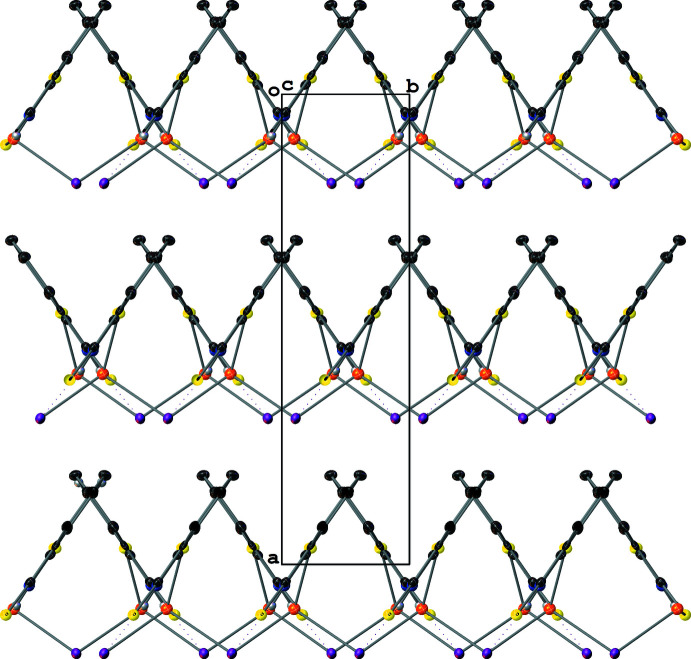
A mol­ecular drawing of **V** viewed along the crystallographic *c* axis with 50% probability ellipsoids. Dotted lines are used to indicate hydrogen-bonding inter­actions. All H atoms bound to C atoms are omitted.

**Figure 13:**
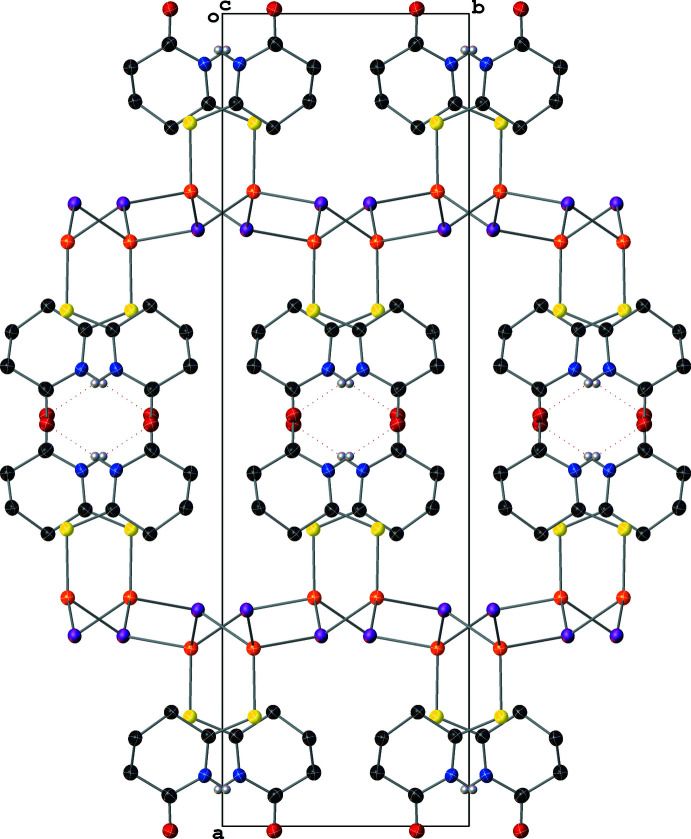
A mol­ecular drawing of **III** viewed along the crystallographic *c* axis with 50% probability ellipsoids. Dotted lines are used to indicate hydrogen-bonding inter­actions. All H atoms bound to C atoms are omitted.

The polymeric sheet propagates parallel to the (100) crystallographic plane. The μ_2_-I^−^ atoms bridge two Cu centers and form Cu–I zigzag chains that propagate parallel to the [010] crystallographic direction. Similarly to **IV**, the μ_2_-SNS6 ligands participate in the polymer propagation in **V** by bridging two Cu atoms and connecting the Cu–I chains and are generated by the *c* glide plane (Fig. 11 ▸). Among the five structures discussed, **V** is the only non-centrosymmetric structure. This results in a packing motif with a polar arrangement of SNS6 ligands on one side of the inorganic sheets, which results in a smaller spacing between the inorganic layers [7.598 (3) Å, see Fig. 12 ▸] in **V** than in **III** [14.134 (5) Å, see Fig. 13 ▸].

## Supra­molecular features   

Among the five structures reported in this work, **III**, **IV**, and **V** crystallize as polymeric sheets; their extended structural characteristics are described above. In addition to the polymeric structural features in **III**, **IV**, and **V**, there are also several types of inter­molecular inter­actions present in each of the five structures that are relevant to a description of their supra­molecular architectures.

All structures except **III** display non-classical (*e.g*., H-atom acceptors that are not N, O or Cl) hydrogen-bonding inter­actions between the N—H of the SNO/SNS/SNS6 ligands and the μ_2_–I^−^/μ_3_–I^−^ atoms. According to our statistical analysis of 3396 N—H⋯I inter­actions observed in 2030 structures reported to the CSD, their *D*⋯*A* distances range from 3.15 to 4.12 Å with a mean *D*⋯*A* distance of 3.69 (13) Å. The *D*⋯*A* distances in structures **I**, **II**, **IV**, and **V** are typical for these types of inter­actions (Table 2 ▸). For structures **I** and **II**, the N—H⋯I inter­action is intra­molecular. For **IV**, there are two symmetry-independent hydrogen–bonding inter­actions, which is expected given that the structure contains two symmetry-independent SNS ligands. The first, between atoms N1—H1⋯I1^ii^ [symmetry code: (ii) −*x* + 1, −*y* + 1, −*z* + 1], is a stronger inter­action; the second is between atoms N2^iv^—H2^iv^⋯I1 [symmetry code: (iv) *x* + 1, *y*, *z*] and is a weaker inter­action (Table 2 ▸). Both inter­actions form *S*(6) hydrogen-bonding motifs (Etter *et al.* 1990 ▸), which provide some rigidity to the mesh-like sheet of the polymer.

Structure **III** is unique among all the structures discussed in this work as it is the only structure to exhibit classical hydrogen-bonding inter­actions. There are two identical hydrogen bonds per SNO ligand, with the N—H serving as an H-bond donor and the O atom serving as an H-bond acceptor [N1—H1⋯O1^iii^ and N1^iii^—H^iii^⋯O1; symmetry code: (iii) −*x* + 1, −*y* + 1, −*z* + 2]. These hydrogen bonds are relatively strong (Table 2 ▸) and form 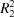
(8) motifs between the stacked [Cu_3_I_3_]_*n*_ polymeric layers. Their presence leads to an extended three-dimensional framework structure, where the propagation of the [Cu_3_I_3_]_*n*_ polymeric sheets accounts for two dimensions and the connection of those sheets through the hydrogen-bonding inter­actions provides the third (Fig. 13 ▸).

Structure **V** has two distinct types of inter­molecular inter­actions. First, there is the non-classical hydrogen-bonding inter­action between the N—H of the SNS6 ligand and the symmetry-equivalent μ_2_-I^−^ atoms [N1—H1⋯I1^ii;^ symmetry code: (ii) *x*, 1 − *y*, −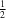 + *z*] within the same polymeric sheet. This inter­action forms 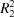
(6) motifs that are of typical strength (see Figs. 10 ▸ and 11 ▸; Table 2 ▸). In addition to the non-classical hydrogen-bonding inter­actions, there are also π–π stacking inter­actions between SNS6 ligands within the same polymeric sheet due to the presence of the extended π system in the SNS6 ligand backbone. These inter­actions, formed by the overlap between the five-membered rings with atoms C1–C2–C7–C8–N1 (*R*_5_) and the phenyl rings with atoms C2^i^–C3^i^–C4^i^–C5^i^–C6^i^–C7^i^ (*R*_6_) [symmetry code: (i) *x*, 1 + *y*, *z*], is of moderate strength [plane *R*_5_ to *R*_6_ centroid distance: 3.369 (5) Å; R_5_ to *R*_6_ centroid offset distance: 1.165 (14) Å]. These π–π stacking inter­actions, in tandem with the increased size of the SNS6 ligand relative to the SNS/SNO ligands, results in a tightly packed two-dimensional sheet (packing coefficient: 71.8%), which prevents the formation of the more mesh-like structure seen in **IV** (packing coefficient: 54.1%) (Kitaigorodskii, 1973 ▸).

## Database survey   

All searches in the Cambridge Structural Database (Version 5.41, latest update May 2020; Groom *et al.* 2016 ▸) were performed with moderate search criteria (for structures **I** and **II**: no errors or ions, not polymeric, only single crystal structures; for structures **III**, **IV**, and **V**: no errors or ions, only single crystal structures. The surveys of the database for each individual structure are described below.

**I**: A search for Cu_2_(μ_2_-I)_2_ dimers with two neutral ligands binding with one nitro­gen and one sulfur atom resulted in 17 matches. Only one had a homometallic [Cu(μ_2_-I)_2_(S)(N)]_2_ type structure where the S and N donors were part of monodentate ligands, which indicates that the coordination environment in **I** is a relatively unusual one. This structure, bis­[(μ_2_-iodo)(aceto­nitrile)(tri­phenyl­thio­phospho­rane)copper(I)] (refcode: OCALOT; Lobana *et al.*, 2001 ▸), has similar Cu—S and Cu—N distances and a slightly longer Cu—I distance. However, OCALOT has a dramatically longer Cu⋯Cu distance [3.4141 (16) Å] than that in **I** (Table 1 ▸). This elongation is likely due to the larger steric requirements of the SPPh_3_ sulfur donor ligand in OCALOT.

**II**: a survey of the Cambridge Structural Database for Cu_4_(μ_3_-I)_4_ tetra­hedrons with a mix of two Cu I_3_N coordination spheres and two I_3_S coordination spheres provided only one match, octa­kis­(μ_3_-iodo)­bis­{μ_2_-bis­[(2,4-di­methyl­phen­yl)thio]­methane-*S*,*S*′}tetra­kis­(aceto­nitrile)­octa­copper(I) aceto­nitrile tetra­hydro­furan solvate (refcode: ENAXAT; Martínez-Alanis *et al.*, 2011 ▸), which features two Cu_4_(μ_3_-I)_4_ tetra­hedrons. Two of the Cu centers in each tetra­hedron have a (μ_3_-I)_3_(NCCH_3_) coordination sphere. The other two Cu centers have (μ_3_-I)_3_S coordination spheres with bridging bis­[(3,5-di­methyl­phen­yl)thio]­methane ligands that tether the two tetra­hedra together. The geometric parameters of this structure [Cu⋯Cu, Cu—I, Cu—S, and Cu—N distances: 2.69 (3), 2.68 (4), 2.315 (11), and Cu—N 1.979 (6) Å] are very similar to those in **II**.

An additional, broader search for all non-polymeric Cu_4_(μ_3_-I) tetra­hedra yielded 130 results for Cu_4_(μ_3_-I)_4_(*L*)_4_ (*L* = N, S, P, I, O, As) tetra­hedra with *L* as a neutral ligand. All of the resulting structures had identical first coordination spheres for each of the Cu centers [*e.g*., Cu_4_(μ_3_-I)_4_(*L*)_4_, rather than the Cu_4_(μ_3_-I)_4_(*L*)_2_(*L*′)_2_ in **II**]. To the best of our knowledge, **II** is the first reported instance of a non-polymeric Cu_4_(μ_3_-I)_4_ tetra­hedron with N and (non-bridging) S ligands.

**III**: A search for structures containing Cu_3_(μ_3_-*X*)_3_ ring motifs that did not contain Cu_4_(μ_3_-*X*)_4_ (*X* = any halogen) tetra­hedral motifs yielded 60 structures. Four of them contained Cu_3_(μ_3_-*X*)_3_ motifs and one of them (DENQEV; Liu *et al.*, 2018 ▸) contained a Cu_3_(μ_3_-*X*)_3_ ring motif. This structure, *catena*-[bis­[μ-5-(1-amino­eth­yl)tetra­zolato]tetra­kis­(μ-iodo)­copper(II)tetra­copper(I)], contains four monovalent and one divalent symmetry-independent Cu centers that form a one-dimensional ribbon. This ribbon, in combination with the bridging (1*S*)-1-(5-tetra­zol­yl) ethyl­amine ligands, forms a three-dimensional network. There are a few other examples of copper halide extended structures based on Cu_3_(μ_3_-*X*)_3_ ring motifs that are both one-dimensional (Näther & Jess, 2003 ▸; Oliver *et al.*, 1977 ▸) and two-dimensional (Blake *et al.*, 1999 ▸; Haakansson *et al.*, 1991 ▸; Haakansson & Jagner, 1990 ▸). Among these, the [Cu_2_I_2_(μ_3_-1,3,5-triazine)]_∞_ structure reported by Blake *et al.* is the only two-dimensional sheet with hexa­gonal Cu_3_I_3_ rings and μ_3_-triazine linkers. To the best of our knowledge, **III** is the first instance of this extended hexa­gonal Cu_3_I_3_ structure that is not supported by bridging ligands.

**IV**: A search for polymeric structures containing Cu_2_(μ_2_-*X*)_2_(μ_2_-S) (*X* = F^−^, Cl^−^, Br^−^, I^−^) rhombi afforded 91 matches that included 35 polymeric homometallic structures. Among the 35 structures, 23 were two-dimensional polymers. Whereas there were no closely related matches for **IV**, a similar structure (JIZPEQ; Raghuvanshi *et al.*, 2019 ▸) was found. This structure has the same chemical composition as **IV** except with μ_2_-1,3-di­thiane ligands rather than μ_2_-SNS ligands. In contrast to the two-dimensional mesh-like structure of **IV**, JIZPEQ crystallizes with one-dimensional chains with links comprised of two Cu_2_(μ_2_-I)_2_ rhombi and two μ_2_-1,3-di­thiane ligands. The geometric parameters of the Cu_2_(μ_2_-I)_2_ rhombi are in good agreement with those in **IV** [Cu⋯Cu, Cu—I (average), and Cu—S (average) distances: 2.8904 (6), 2.63 (2), and 2.329 (3) Å].

**V**: A search for structures with Cu—*X* zigzag chains that did not contain the Cu_2_(μ_2_-*X*)_2_ rhombus afforded 112 matches. Among these, 56 were for polymeric homometallic structures and three of these [refcodes: AFUDUA (Caradoc-Davies *et al.*, 2002 ▸), CIQQOL (Musina *et al.*, 2017 ▸), and FIWWAK (Cingolani *et al.*, 2005 ▸)] contained one-dimensional Cu—I^−^ zigzag chains. All three structures contain tetra­hedral Cu^I^ centers coordinated by the two μ_2_-I atoms and two neutral donor ligands (binding with sulfur and nitro­gen for AFUNDA, arsenic for CIQQOL, and FIWWAK). These structures have similar geometries to that of **V** except for the Cu–ligand distances.

## Synthesis and crystallization   

The ligands piperidine-2,6-di­thione (SNS) and 6-thioxopiperidin-2-one (SNO) were purchased from Sigma–Aldrich and used as received. Isoindoline-1,3-di­thione (SNS6) was prepared in a similar manner to that previously described in the literature (Yde *et al.*, 1984 ▸).

Unless otherwise specified, all reactions were performed at room temperature under a dry N_2_ atmosphere using standard glovebox methods.

**I** was prepared by combining 10 ml of a clear yellow solution of 6-thioxopiperidin-2-one (0.500 mmol) in di­chloro­methane with 10 mL of a colorless solution of CuI (0.502 mmol) in aceto­nitrile. Upon combination, the solution turned a bright-orange color. Vapor diffusion of the orange solution with diethyl ether afforded large, yellow, block-shaped crystals of **I** after three days.

Two by-products were also obtained from the reaction of 6-thioxopiperidin-2-one and CuI. The first (**II**) were small, yellow, plate-shaped crystals that co-crystallized with the larger yellow block-shaped crystals of **I**. The second by-product formed after exposing the initial orange solution from the reaction of 6-thioxopiperidin-2-one and CuI to air, and allowing that solution to slowly evaporate in air for approximately one week. After this time, small, red–orange crystals of **III** were obtained.

**IV** was prepared by layering 10 mL of a clear yellow solution of piperidine-2,6-di­thione (1.01 mmol) in di­chloro­methane over 10 mL of a colorless solution of CuI (1.00 mmol) in aceto­nitrile. Dark-red crystals of **IV** were obtained after one week.

Black, needle-shaped crystals of **V** were obtained in a similar manner to **IV**, with the exception that 1.00 mmol of isoindoline-1,3-di­thione was used instead of piperidine-2,6-di­thione.

## Refinement   

For structure **I**, the diffraction data were consistent with the space groups *P*1 and *P* 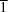; the *E*-statistics were consistent for the centrosymmetric space group *P* 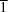 and were used to make the final space-group determination. For structures **II**–**V**, a combination of the systematic absences in the diffraction data and the *E*-statistics were used to assign the centrosymmetric space groups *C*2/*c* (**II**), *Pbcn* (**III**), *P*2_1_/*c* (**IV**) and the non-centrosymmetric space group *Cc* (**V**).

The structures were solved *via* intrinsic phasing and refined by least-squares refinement on *F* ^2^ followed by difference-Fourier synthesis. All non-hydrogen atoms were refined with anisotropic displacement parameters. Unless otherwise specified, all hydrogen atoms were included in the final structure-factor calculation at idealized positions and were allowed to ride on the neighboring atoms with relative isotropic displacement coefficients.

The coordinates of the H atoms bound to N atoms in structures **I**, **II**, and **III** were refined freely with a distance restraint for each N—H distance.

In structure **IV**, there were three partially occupied solvent mol­ecules of di­chloro­methane and one partially occupied mol­ecule of aceto­nitrile present in the asymmetric unit. A significant amount of time was invested in identifying and refining the disordered mol­ecules. Bond-length restraints were applied to model the mol­ecules, but the resulting isotropic displacement coefficients suggested the mol­ecules were mobile. In addition, the refinement was computationally unstable. The SQUEEZE option (Spek, 2015 ▸) of the *PLATON* software suite (Spek, 2020 ▸) was used to correct the diffraction data for diffuse scattering effects and to identify the solvent mol­ecule. *PLATON* calculated the upper limit of volume that can be occupied by the solvent in the unit cell to be 615 Å^3^. This solvent-accessible volume is comprised of two smaller (*ca* 115 Å^3^) and two larger (*ca* 196 Å^3^) solvent-accessible voids and is 27% of the unit-cell volume. The program calculated 155 electrons in the unit cell for the diffuse species. This corresponds to approximately one mol­ecule of di­chloro­methane (42 electrons) that is 50% occupied and one mol­ecule of aceto­nitrile (22 electrons) in the asymmetric unit. It is very likely that the solvent mol­ecules are disordered over several positions. All derived results in Tables 1 ▸ and 2 ▸ are based on the known contents. No data are given for the diffusely scattering species.

Crystal data, data collection and structure refinement details are summarized in Table 3 ▸.

## Supplementary Material

Crystal structure: contains datablock(s) I, II, III, IV, V. DOI: 10.1107/S2056989020009676/zl2791sup1.cif

Structure factors: contains datablock(s) I. DOI: 10.1107/S2056989020009676/zl2791Isup7.hkl

Structure factors: contains datablock(s) II. DOI: 10.1107/S2056989020009676/zl2791IIsup8.hkl

Structure factors: contains datablock(s) III. DOI: 10.1107/S2056989020009676/zl2791IIIsup9.hkl

Structure factors: contains datablock(s) IV. DOI: 10.1107/S2056989020009676/zl2791IVsup10.hkl

Structure factors: contains datablock(s) V. DOI: 10.1107/S2056989020009676/zl2791Vsup11.hkl

CCDC references: 2016675, 2016674, 2016673, 2016672, 2016671

Additional supporting information:  crystallographic information; 3D view; checkCIF report

## Figures and Tables

**Table 1:** Selected bond lengths for structures **I**–**V**

	**I** *^*a*^*		**II** *^*b*^*		**III** *^*c*^*		**IV** *^*d*^*		**V** *^*e*^*	
Cu—I	I1—Cu1	2.6261 (6)	I1—Cu1	2.6451 (6)	I1—Cu1	2.6264 (11)	I1—Cu1	2.6365 (8)	I1—Cu1	2.6152 (13)
	I1—Cu1^i^	2.6321 (7)	I1—Cu2^i^	2.7017 (7)	I1—Cu1^i^	2.6709 (12)	I1—Cu1^i^	2.6687 (8)	I1—Cu1^i^	2.6798 (13)
			I1—Cu2	2.7250 (6)	I1—Cu1^ii^	2.6342 (10)	I2—Cu2	2.6719 (8)		
			I2—Cu1	2.7796 (6)			I2—Cu2^ii^	2.6724 (8)		
			I2—Cu1^i^	2.6542 (6)						
			I2—Cu2	2.6456 (6)						
Cu⋯Cu	Cu1—Cu1^i^	2.7274 (6)	Cu1—Cu1^i^	2.8150 (11)						
			Cu1—Cu2	2.7864 (8)						
			Cu1—Cu2^i^	2.7106 (8)						
			Cu2—Cu2^i^	2.5803 (10)						
Cu—S	Cu1—S1	2.3205 (6)	Cu1–S1	2.2869 (10)	Cu1—S1	2.2827 (15)	Cu1—S1	2.3086 (14)	Cu1—S1	2.269 (2)
							Cu1—S4^iii^	2.3075 (13)	Cu1—S2^ii^	2.273 (2)
							Cu2—S2	2.2802 (15)		
							Cu2—S3	2.2933 (15)		
Cu—N	Cu1—N2	2.0225 (10)	Cu2—N2	1.974 (3)						

Symmetry codes: (*a*) (i) −*x* + 1, −*y* + 1, −*z* for **I**; (*b*) (i) −*x* + 1, *y*, −*z* + 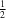 for **II**; (*c*) (i) *-*x** + 1/2, −*y* + 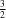, *z* − 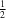 and (ii) *x*, −*y* + 1, *z* − 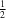 for **III**; (*d*) (i) −*x*, −*y*, −*z* + 1; (ii) −*x* + 1, −*y* + 1, −*z* + 1 and (iii) −*x* + 1, −*y*, −*z* + 1 for **IV**; (*e*) (i) *x*, *y* − 1, *z* and (ii) *x*, −*y* + 1, *z* + 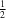 for **V**.

**Table 2:** Hydrogen bonding geometries for **I**–**V**

	*D*—H⋯*A*	*D*—H	H⋯*A*	*D*⋯*A*	*D*—H⋯*A*
**I** *^*a*^*	N1—H1⋯I1^i^	0.857 (12)	2.845 (13)	3.6980 (12)	173.8 (13)
**II**	N1—H1⋯I2	0.870 (19)	2.81 (2)	3.672 (3)	170 (4)
**III** *^*b*^*	N1—H1⋯O1^iii^	0.86 (2)	2.03 (2)	2.881 (5)	171 (5)
**IV** *^*c*^*	N1—H1⋯I2^ii^	0.88	2.79	3.628 (4)	160.9
	N2—H2⋯I1^iv^	0.88	2.90	3.679 (4)	149.2
**V** *^*d*^*	N1—H1⋯I1^iii^	0.88	2.84	3.692 (7)	163.2

Symmetry codes: (*a*) (i) −*x* + 1, −*y* + 1, −*z* for **I**; (*b*) (iii) −*x* + 1, −*y* + 1, −*z* + 2 for **III**; (*c*) (ii) −*x* + 1, −*y* + 1, −*z* + 1 and (iv) *x* + 1, *y*, *z* for **IV**; (*d*) (iii) *x*, −*y* + 1, *z* − 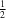 for **IV**.

**Table 3:** Experimental details

	**I**	**II**	**III**	**IV**	**V**
Crystal data					
Chemical formula	[Cu_2_I_2_(C_2_H_3_N)_2_(C_5_H_7_NOS)_2_]	[Cu_4_I_4_(C_2_H_3_N)_2_(C_5_H_7_NOS)_2_]	[CuI(C_5_H_7_NOS)]	[Cu_2_I_2_(C_5_H_7_NS_2_)_2_]	[CuI(C_8_H_5_NS_2_)]
*M*_r_	721.34	1102.22	319.62	671.35	369.69
Crystal system, space group	Triclinic, *P* 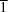	Monoclinic, *C*2/*c*	Orthorhombic, *P* *b* *c* *n*	Monoclinic, *P*2_1_/*c*	Monoclinic, *C* *c*
Temperature (K)	100	105	100	100	100
*a*, *b*, *c* (Å)	8.121 (2), 8.433 (2), 9.154 (2)	14.4669 (8), 12.2157 (7), 16.9969 (11)	26.982 (11), 8.195 (4), 7.351 (3)	13.2866 (9), 11.6974 (13), 14.8089 (9)	15.174 (5), 4.1188 (16), 15.785 (6)
α, β, γ (°)	68.918 (12), 80.523 (12), 71.270 (9)	90, 112.562 (5), 90	90, 90, 90	90, 96.998 (6), 90	90, 92.98 (2), 90
*V* (Å^3^)	553.2 (2)	2773.9 (3)	1625.4 (13)	2284.4 (3)	985.2 (6)
*Z*	1	4	8	4	4
Radiation type	Mo *K*α	Cu *K*α	Cu *K*α	Cu *K*α	Mo *K*α
μ (mm^−1^)	4.92	39.97	35.47	26.87	5.72
Crystal size (mm)	0.15 × 0.13 × 0.13	0.1 × 0.08 × 0.04	0.08 × 0.07 × 0.03	0.22 × 0.13 × 0.09	0.30 × 0.02 × 0.01

Data collection					
Diffractometer	Bruker APEXII Quazar	Bruker SMART APEXII area detector	Bruker SMART APEXII area detector	Bruker SMART APEXII area detector	Bruker APEXII Quazar
Absorption correction	Multi-scan (*SADABS*; Bruker, 2016 ▸)	Multi-scan (*SADABS*; Bruker, 2016 ▸)	Multi-scan *SADABS*; Bruker, 2016 ▸)	Multi-scan (*SADABS*; Bruker, 2016 ▸)	Multi-scan (*SADABS*; Bruker, 2016 ▸)
*T*_min_, *T*_max_	0.079, 0.120	0.042, 0.188	0.030, 0.144	0.009, 0.094	0.322, 0.404
No. of measured, independent and observed [*I* > 2σ(*I*)] reflections	17922, 4099, 3988	23553, 2753, 2669	25274, 1631, 1467	78133, 4597, 4236	11871, 3603, 3226
*R*_int_	0.021	0.060	0.051	0.063	0.041
(sin θ/λ)_max_ (Å^−1^)	0.779	0.622	0.622	0.622	0.770

Refinement					
*R*[*F* ^2^ > 2σ(*F* ^2^)], *wR*(*F* ^2^), *S*	0.012, 0.030, 1.07	0.026, 0.063, 1.10	0.032, 0.082, 1.07	0.048, 0.126, 1.06	0.036, 0.089, 1.06
No. of reflections	4099	2753	1631	4597	3603
No. of parameters	122	140	94	181	118
No. of restraints	1	1	1	0	2
H-atom treatment	H atoms treated by a mixture of independent and constrained refinement	H atoms treated by a mixture of independent and constrained refinement	H atoms treated by a mixture of independent and constrained refinement	H-atom parameters constrained	H-atom parameters constrained
Δρ_max_, Δρ_min_ (e Å^−3^)	0.43, −0.34	0.84, −1.25	1.31, −1.03	1.58, −0.49	3.17, −1.13
Absolute structure	–	–	–	–	Flack *x* determined using 1438 quotients [(*I* ^+^)−(*I* ^−^)]/[(*I* ^+^)+(*I* ^−^)] (Parsons *et al.*, 2013 ▸)
Absolute structure parameter	–	–	–	–	0.034 (12)

Computer programs: *APEX3* (Bruker, 2017 ▸), *APEX2* and *SAINT* (Bruker, 2013 ▸), *SHELXT* (Sheldrick, 2015*a* ▸), *SHELXL* (Sheldrick, 2015*b* ▸), and *OLEX2* (Dolomanov *et al.*, 2009 ▸).

## supplementary crystallographic information

### Di-µ-iodido-bis[(acetonitrile-κN)(6-sulfanylidenepiperidin-2-one-κS)copper(I)] (I) Crystal data

**Table:**

[Cu_2_I_2_(C_2_H_3_N)_2_(C_5_H_7_NOS)_2_]	*Z* = 1
*M**_r_* = 721.34	*F*(000) = 344
Triclinic, *P*1	*D*_x_ = 2.165 Mg m^−^^3^
*a* = 8.121 (2) Å	Mo *K*α radiation, λ = 0.71073 Å
*b* = 8.433 (2) Å	Cell parameters from 9905 reflections
*c* = 9.154 (2) Å	θ = 2.4–33.6°
α = 68.918 (12)°	µ = 4.92 mm^−^^1^
β = 80.523 (12)°	*T* = 100 K
γ = 71.270 (9)°	Block, yellow
*V* = 553.2 (2) Å^3^	0.15 × 0.13 × 0.13 mm

### Di-µ-iodido-bis[(acetonitrile-κN)(6-sulfanylidenepiperidin-2-one-κS)copper(I)] (I) Data collection

**Table:**

Bruker APEXII Quazar diffractometer	4099 independent reflections
Radiation source: microfocus sealed X-ray tube, Incoatec Iµs	3988 reflections with *I* > 2σ(*I*)
Mirror optics monochromator	*R*_int_ = 0.021
Detector resolution: 7.9 pixels mm^-1^	θ_max_ = 33.6°, θ_min_ = 2.4°
0.5° ω and 0.5° φ scans	*h* = −12→12
Absorption correction: multi-scan (SADABS; Bruker, 2016)	*k* = −12→13
*T*_min_ = 0.079, *T*_max_ = 0.120	*l* = −14→13
17922 measured reflections	

### Di-µ-iodido-bis[(acetonitrile-κN)(6-sulfanylidenepiperidin-2-one-κS)copper(I)] (I) Refinement

**Table:**

Refinement on *F*^2^	Primary atom site location: dual
Least-squares matrix: full	Hydrogen site location: mixed
*R*[*F*^2^ > 2σ(*F*^2^)] = 0.012	H atoms treated by a mixture of independent and constrained refinement
*wR*(*F*^2^) = 0.030	*w* = 1/[σ^2^(*F*_o_^2^) + (0.010*P*)^2^ + 0.2*P*] where *P* = (*F*_o_^2^ + 2*F*_c_^2^)/3
*S* = 1.07	(Δ/σ)_max_ = 0.001
4099 reflections	Δρ_max_ = 0.43 e Å^−^^3^
122 parameters	Δρ_min_ = −0.34 e Å^−^^3^
1 restraint	

### Di-µ-iodido-bis[(acetonitrile-κN)(6-sulfanylidenepiperidin-2-one-κS)copper(I)] (I) Special details

**Table:**

Geometry. All esds (except the esd in the dihedral angle between two l.s. planes) are estimated using the full covariance matrix. The cell esds are taken into account individually in the estimation of esds in distances, angles and torsion angles; correlations between esds in cell parameters are only used when they are defined by crystal symmetry. An approximate (isotropic) treatment of cell esds is used for estimating esds involving l.s. planes.

### Di-µ-iodido-bis[(acetonitrile-κN)(6-sulfanylidenepiperidin-2-one-κS)copper(I)] (I) Fractional atomic coordinates and isotropic or equivalent isotropic displacement parameters (Å^2^)

**Table:**

	*x*	*y*	*z*	*U*_iso_*/*U*_eq_	
I1	0.71563 (2)	0.27834 (2)	−0.05209 (2)	0.01894 (2)	
Cu1	0.45123 (2)	0.38128 (2)	0.13294 (2)	0.01894 (3)	
S1	0.56399 (3)	0.24010 (4)	0.37914 (3)	0.01986 (5)	
O1	0.05297 (11)	0.66482 (10)	0.51701 (10)	0.02183 (14)	
N1	0.28752 (11)	0.45357 (12)	0.47278 (10)	0.01722 (15)	
H1	0.278 (2)	0.5149 (19)	0.3753 (15)	0.021*	
N2	0.26648 (13)	0.25871 (13)	0.15713 (11)	0.02214 (17)	
C1	0.41889 (13)	0.30218 (13)	0.51318 (11)	0.01629 (16)	
C2	0.42947 (14)	0.19443 (14)	0.68381 (12)	0.02105 (18)	
H2A	0.508966	0.227581	0.731337	0.025*	
H2B	0.479000	0.067327	0.694709	0.025*	
C3	0.25216 (15)	0.22197 (14)	0.77191 (12)	0.02196 (19)	
H3A	0.265859	0.157362	0.885379	0.026*	
H3B	0.176642	0.174405	0.734521	0.026*	
C4	0.16790 (15)	0.41932 (14)	0.74515 (12)	0.02137 (19)	
H4A	0.048628	0.437280	0.795116	0.026*	
H4B	0.235816	0.462532	0.795254	0.026*	
C5	0.15956 (13)	0.52448 (13)	0.57383 (12)	0.01738 (17)	
C6	0.17193 (13)	0.17425 (13)	0.20890 (12)	0.01823 (17)	
C7	0.05487 (15)	0.06411 (15)	0.27580 (14)	0.02279 (19)	
H7A	0.012545	0.043830	0.191652	0.034*	
H7B	−0.044124	0.123970	0.332437	0.034*	
H7C	0.117167	−0.049821	0.348452	0.034*	

### Di-µ-iodido-bis[(acetonitrile-κN)(6-sulfanylidenepiperidin-2-one-κS)copper(I)] (I) Atomic displacement parameters (Å^2^)

**Table:**

	*U*^11^	*U*^22^	*U*^33^	*U*^12^	*U*^13^	*U*^23^
I1	0.02097 (3)	0.01735 (3)	0.01597 (3)	−0.00396 (2)	0.00281 (2)	−0.00554 (2)
Cu1	0.02026 (6)	0.01995 (6)	0.01757 (6)	−0.00817 (5)	0.00300 (4)	−0.00688 (5)
S1	0.01667 (10)	0.02251 (11)	0.01925 (11)	−0.00292 (9)	0.00063 (8)	−0.00856 (9)
O1	0.0229 (4)	0.0176 (3)	0.0234 (4)	−0.0036 (3)	0.0001 (3)	−0.0073 (3)
N1	0.0191 (4)	0.0168 (4)	0.0141 (3)	−0.0045 (3)	0.0007 (3)	−0.0044 (3)
N2	0.0219 (4)	0.0223 (4)	0.0229 (4)	−0.0073 (3)	0.0004 (3)	−0.0079 (3)
C1	0.0164 (4)	0.0175 (4)	0.0165 (4)	−0.0059 (3)	−0.0013 (3)	−0.0062 (3)
C2	0.0237 (5)	0.0204 (4)	0.0161 (4)	−0.0028 (4)	−0.0038 (3)	−0.0044 (3)
C3	0.0292 (5)	0.0181 (4)	0.0165 (4)	−0.0082 (4)	0.0017 (4)	−0.0033 (3)
C4	0.0270 (5)	0.0200 (4)	0.0159 (4)	−0.0075 (4)	0.0037 (4)	−0.0059 (3)
C5	0.0192 (4)	0.0169 (4)	0.0176 (4)	−0.0071 (3)	0.0020 (3)	−0.0071 (3)
C6	0.0180 (4)	0.0177 (4)	0.0183 (4)	−0.0029 (3)	−0.0013 (3)	−0.0069 (3)
C7	0.0233 (5)	0.0219 (5)	0.0254 (5)	−0.0108 (4)	0.0042 (4)	−0.0088 (4)

### Di-µ-iodido-bis[(acetonitrile-κN)(6-sulfanylidenepiperidin-2-one-κS)copper(I)] (I) Geometric parameters (Å, º)

**Table:**

I1—Cu1	2.6261 (6)	C2—H2B	0.9900
I1—Cu1^i^	2.6321 (7)	C2—C3	1.5216 (16)
Cu1—Cu1^i^	2.7274 (6)	C3—H3A	0.9900
Cu1—S1	2.3205 (6)	C3—H3B	0.9900
Cu1—N2	2.0225 (10)	C3—C4	1.5260 (16)
S1—C1	1.6607 (11)	C4—H4A	0.9900
O1—C5	1.2117 (13)	C4—H4B	0.9900
N1—H1	0.857 (12)	C4—C5	1.4988 (15)
N1—C1	1.3493 (13)	C6—C7	1.4518 (15)
N1—C5	1.4055 (13)	C7—H7A	0.9800
N2—C6	1.1446 (14)	C7—H7B	0.9800
C1—C2	1.4987 (15)	C7—H7C	0.9800
C2—H2A	0.9900		
			
Cu1—I1—Cu1^i^	62.489 (13)	C3—C2—H2B	109.2
I1—Cu1—I1^i^	117.511 (13)	C2—C3—H3A	109.7
I1^i^—Cu1—Cu1^i^	58.647 (16)	C2—C3—H3B	109.7
I1—Cu1—Cu1^i^	58.864 (17)	C2—C3—C4	109.71 (9)
S1—Cu1—I1	102.61 (2)	H3A—C3—H3B	108.2
S1—Cu1—I1^i^	118.719 (16)	C4—C3—H3A	109.7
S1—Cu1—Cu1^i^	132.375 (17)	C4—C3—H3B	109.7
N2—Cu1—I1^i^	105.18 (3)	C3—C4—H4A	109.3
N2—Cu1—I1	111.38 (3)	C3—C4—H4B	109.3
N2—Cu1—Cu1^i^	127.14 (3)	H4A—C4—H4B	108.0
N2—Cu1—S1	100.19 (3)	C5—C4—C3	111.49 (9)
C1—S1—Cu1	109.94 (4)	C5—C4—H4A	109.3
C1—N1—H1	118.1 (10)	C5—C4—H4B	109.3
C1—N1—C5	127.15 (9)	O1—C5—N1	118.17 (9)
C5—N1—H1	114.7 (10)	O1—C5—C4	125.23 (10)
C6—N2—Cu1	162.96 (9)	N1—C5—C4	116.60 (9)
N1—C1—S1	121.16 (8)	N2—C6—C7	178.87 (12)
N1—C1—C2	117.20 (9)	C6—C7—H7A	109.5
C2—C1—S1	121.63 (8)	C6—C7—H7B	109.5
C1—C2—H2A	109.2	C6—C7—H7C	109.5
C1—C2—H2B	109.2	H7A—C7—H7B	109.5
C1—C2—C3	112.14 (9)	H7A—C7—H7C	109.5
H2A—C2—H2B	107.9	H7B—C7—H7C	109.5
C3—C2—H2A	109.2		
			
Cu1—S1—C1—N1	−20.58 (9)	C1—C2—C3—C4	−54.31 (12)
Cu1—S1—C1—C2	160.32 (7)	C2—C3—C4—C5	54.18 (12)
S1—C1—C2—C3	−153.63 (8)	C3—C4—C5—O1	153.16 (10)
N1—C1—C2—C3	27.24 (13)	C3—C4—C5—N1	−27.45 (13)
C1—N1—C5—O1	178.41 (10)	C5—N1—C1—S1	−177.90 (8)
C1—N1—C5—C4	−1.02 (15)	C5—N1—C1—C2	1.24 (15)

Symmetry code: (i) −*x*+1, −*y*+1, −*z*.

### Di-µ-iodido-bis[(acetonitrile-κN)(6-sulfanylidenepiperidin-2-one-κS)copper(I)] (I) Hydrogen-bond geometry (Å, º)

**Table:**

*D*—H···*A*	*D*—H	H···*A*	*D*···*A*	*D*—H···*A*
N1—H1···I1^i^	0.86 (1)	2.85 (1)	3.6980 (12)	174 (1)

Symmetry code: (i) −*x*+1, −*y*+1, −*z*.

### Bis(acetonitrile-κN)tetra-µ3-iodido-bis(6-sulfanylidenepiperidin-2-one-κS)-tetrahedro-tetracopper(I) (II) Crystal data

**Table:**

[Cu_4_I_4_(C_2_H_3_N)_2_(C_5_H_7_NOS)_2_]	*F*(000) = 2032
*M**_r_* = 1102.22	*D*_x_ = 2.639 Mg m^−^^3^
Monoclinic, *C*2/*c*	Cu *K*α radiation, λ = 1.54178 Å
*a* = 14.4669 (8) Å	Cell parameters from 9916 reflections
*b* = 12.2157 (7) Å	θ = 4.9–73.4°
*c* = 16.9969 (11) Å	µ = 39.97 mm^−^^1^
β = 112.562 (5)°	*T* = 105 K
*V* = 2773.9 (3) Å^3^	Block, yellow
*Z* = 4	0.1 × 0.08 × 0.04 mm

### Bis(acetonitrile-κN)tetra-µ3-iodido-bis(6-sulfanylidenepiperidin-2-one-κS)-tetrahedro-tetracopper(I) (II) Data collection

**Table:**

Bruker SMART APEXII area detector diffractometer	2753 independent reflections
Radiation source: sealed X-ray tube, Siemens, K FFCU 2K 90	2669 reflections with *I* > 2σ(*I*)
Equatorially mounted graphite monochromator	*R*_int_ = 0.060
Detector resolution: 7.9 pixels mm^-1^	θ_max_ = 73.4°, θ_min_ = 4.9°
0.60° ω and 0.6° φ scans	*h* = −17→17
Absorption correction: multi-scan (SADABS; Bruker, 2016)	*k* = −15→15
*T*_min_ = 0.042, *T*_max_ = 0.188	*l* = −19→20
23553 measured reflections	

### Bis(acetonitrile-κN)tetra-µ3-iodido-bis(6-sulfanylidenepiperidin-2-one-κS)-tetrahedro-tetracopper(I) (II) Refinement

**Table:**

Refinement on *F*^2^	Primary atom site location: dual
Least-squares matrix: full	Hydrogen site location: mixed
*R*[*F*^2^ > 2σ(*F*^2^)] = 0.026	H atoms treated by a mixture of independent and constrained refinement
*wR*(*F*^2^) = 0.063	*w* = 1/[σ^2^(*F*_o_^2^) + (0.0336*P*)^2^ + 8.1228*P*] where *P* = (*F*_o_^2^ + 2*F*_c_^2^)/3
*S* = 1.10	(Δ/σ)_max_ = 0.001
2753 reflections	Δρ_max_ = 0.84 e Å^−^^3^
140 parameters	Δρ_min_ = −1.25 e Å^−^^3^
1 restraint	

### Bis(acetonitrile-κN)tetra-µ3-iodido-bis(6-sulfanylidenepiperidin-2-one-κS)-tetrahedro-tetracopper(I) (II) Special details

**Table:**

Geometry. All esds (except the esd in the dihedral angle between two l.s. planes) are estimated using the full covariance matrix. The cell esds are taken into account individually in the estimation of esds in distances, angles and torsion angles; correlations between esds in cell parameters are only used when they are defined by crystal symmetry. An approximate (isotropic) treatment of cell esds is used for estimating esds involving l.s. planes.

### Bis(acetonitrile-κN)tetra-µ3-iodido-bis(6-sulfanylidenepiperidin-2-one-κS)-tetrahedro-tetracopper(I) (II) Fractional atomic coordinates and isotropic or equivalent isotropic displacement parameters (Å^2^)

**Table:**

	*x*	*y*	*z*	*U*_iso_*/*U*_eq_	
I1	0.60668 (2)	0.86418 (2)	0.17606 (2)	0.01452 (8)	
I2	0.64470 (2)	0.62302 (2)	0.36802 (2)	0.01064 (8)	
Cu1	0.56033 (4)	0.66150 (4)	0.20182 (3)	0.01367 (13)	
Cu2	0.57587 (4)	0.82337 (4)	0.32174 (3)	0.01552 (13)	
S1	0.60446 (7)	0.54868 (7)	0.11461 (5)	0.01569 (19)	
O1	0.6145 (2)	0.2772 (2)	0.32682 (16)	0.0174 (6)	
N1	0.6055 (2)	0.3917 (3)	0.22074 (19)	0.0126 (6)	
H1	0.606 (4)	0.445 (3)	0.255 (2)	0.015*	
N2	0.6525 (3)	0.9386 (3)	0.4015 (2)	0.0169 (7)	
C1	0.6091 (3)	0.4194 (3)	0.1448 (2)	0.0123 (7)	
C2	0.6188 (3)	0.3267 (3)	0.0903 (2)	0.0159 (8)	
H2A	0.690589	0.314522	0.102177	0.019*	
H2B	0.585384	0.347436	0.029590	0.019*	
C3	0.5734 (3)	0.2208 (3)	0.1056 (2)	0.0202 (8)	
H3A	0.587416	0.160825	0.072514	0.024*	
H3B	0.499919	0.228893	0.086154	0.024*	
C4	0.6177 (3)	0.1928 (3)	0.2001 (3)	0.0191 (8)	
H4A	0.581117	0.129536	0.210478	0.023*	
H4B	0.688502	0.170761	0.216276	0.023*	
C5	0.6128 (3)	0.2859 (3)	0.2552 (2)	0.0132 (7)	
C6	0.6848 (3)	1.0148 (3)	0.4409 (2)	0.0148 (7)	
C7	0.7242 (3)	1.1137 (3)	0.4894 (2)	0.0180 (8)	
H7A	0.709649	1.176523	0.450663	0.027*	
H7B	0.796763	1.106656	0.519796	0.027*	
H7C	0.692732	1.124854	0.530632	0.027*	

### Bis(acetonitrile-κN)tetra-µ3-iodido-bis(6-sulfanylidenepiperidin-2-one-κS)-tetrahedro-tetracopper(I) (II) Atomic displacement parameters (Å^2^)

**Table:**

	*U*^11^	*U*^22^	*U*^33^	*U*^12^	*U*^13^	*U*^23^
I1	0.01753 (14)	0.01161 (13)	0.01566 (13)	−0.00092 (8)	0.00776 (10)	0.00418 (7)
I2	0.01430 (13)	0.01007 (13)	0.00576 (12)	0.00111 (7)	0.00185 (9)	0.00183 (7)
Cu1	0.0197 (3)	0.0114 (3)	0.0103 (2)	0.0035 (2)	0.0062 (2)	0.00158 (19)
Cu2	0.0167 (3)	0.0119 (3)	0.0134 (2)	−0.0027 (2)	0.0007 (2)	−0.00354 (19)
S1	0.0246 (5)	0.0150 (4)	0.0113 (4)	0.0070 (3)	0.0112 (4)	0.0050 (3)
O1	0.0214 (14)	0.0183 (13)	0.0118 (11)	−0.0011 (11)	0.0056 (11)	0.0035 (10)
N1	0.0198 (17)	0.0107 (14)	0.0071 (13)	0.0003 (12)	0.0049 (12)	−0.0007 (11)
N2	0.0173 (16)	0.0157 (16)	0.0139 (14)	−0.0037 (13)	0.0019 (12)	−0.0027 (13)
C1	0.0123 (17)	0.0152 (18)	0.0078 (14)	0.0021 (13)	0.0021 (13)	0.0000 (13)
C2	0.0216 (19)	0.0150 (18)	0.0118 (15)	0.0062 (15)	0.0071 (15)	−0.0020 (13)
C3	0.023 (2)	0.019 (2)	0.0190 (18)	−0.0007 (16)	0.0083 (16)	−0.0087 (15)
C4	0.027 (2)	0.0127 (18)	0.0225 (18)	−0.0004 (15)	0.0145 (17)	−0.0027 (15)
C5	0.0111 (17)	0.0135 (17)	0.0132 (16)	−0.0019 (13)	0.0029 (14)	−0.0001 (13)
C6	0.0169 (18)	0.0143 (18)	0.0091 (14)	0.0025 (14)	0.0003 (14)	0.0011 (14)
C7	0.029 (2)	0.0071 (17)	0.0103 (16)	−0.0010 (15)	−0.0003 (15)	−0.0020 (13)

### Bis(acetonitrile-κN)tetra-µ3-iodido-bis(6-sulfanylidenepiperidin-2-one-κS)-tetrahedro-tetracopper(I) (II) Geometric parameters (Å, º)

**Table:**

I1—Cu1	2.6451 (6)	N1—C5	1.405 (5)
I1—Cu2^i^	2.7017 (7)	N2—C6	1.137 (5)
I1—Cu2	2.7250 (6)	C1—C2	1.504 (5)
I2—Cu1	2.6542 (6)	C2—H2A	0.9900
I2—Cu1^i^	2.7796 (6)	C2—H2B	0.9900
I2—Cu2	2.6456 (6)	C2—C3	1.518 (6)
Cu1—Cu1^i^	2.8150 (11)	C3—H3A	0.9900
Cu1—Cu2	2.7864 (8)	C3—H3B	0.9900
Cu1—Cu2^i^	2.7106 (8)	C3—C4	1.523 (5)
Cu2—Cu2^i^	2.5803 (10)	C4—H4A	0.9900
Cu1—S1	2.2869 (10)	C4—H4B	0.9900
Cu2—N2	1.974 (3)	C4—C5	1.492 (5)
S1—C1	1.654 (4)	C6—C7	1.451 (5)
O1—C5	1.213 (5)	C7—H7A	0.9800
N1—H1	0.870 (19)	C7—H7B	0.9800
N1—C1	1.354 (5)	C7—H7C	0.9800
			
Cu1—I1—Cu2^i^	60.911 (18)	N2—Cu2—I1^i^	98.80 (11)
Cu1—I1—Cu2	62.493 (17)	N2—Cu2—I1	104.44 (10)
Cu2^i^—I1—Cu2	56.78 (2)	N2—Cu2—I2	114.05 (10)
Cu1—I2—Cu1^i^	62.35 (2)	N2—Cu2—Cu1	151.07 (11)
Cu2—I2—Cu1	63.439 (17)	N2—Cu2—Cu1^i^	143.75 (11)
Cu2—I2—Cu1^i^	59.892 (17)	N2—Cu2—Cu2^i^	134.36 (10)
I1—Cu1—I2	107.43 (2)	C1—S1—Cu1	111.24 (13)
I1—Cu1—I2^i^	112.60 (2)	C1—N1—H1	117 (3)
I1—Cu1—Cu1^i^	110.454 (13)	C1—N1—C5	127.1 (3)
I1—Cu1—Cu2^i^	60.576 (18)	C5—N1—H1	115 (3)
I1—Cu1—Cu2	60.157 (18)	C6—N2—Cu2	169.7 (3)
I2—Cu1—I2^i^	113.84 (2)	N1—C1—S1	121.6 (3)
I2—Cu1—Cu1^i^	61.007 (19)	N1—C1—C2	116.4 (3)
I2^i^—Cu1—Cu1^i^	56.64 (2)	C2—C1—S1	122.0 (3)
I2^i^—Cu1—Cu2	101.87 (2)	C1—C2—H2A	109.0
I2—Cu1—Cu2^i^	107.32 (2)	C1—C2—H2B	109.0
I2—Cu1—Cu2	58.129 (16)	C1—C2—C3	112.8 (3)
Cu2^i^—Cu1—I2^i^	57.602 (18)	H2A—C2—H2B	107.8
Cu2^i^—Cu1—Cu1^i^	60.53 (2)	C3—C2—H2A	109.0
Cu2—Cu1—Cu1^i^	57.881 (18)	C3—C2—H2B	109.0
Cu2^i^—Cu1—Cu2	55.97 (2)	C2—C3—H3A	109.7
S1—Cu1—I1	107.77 (3)	C2—C3—H3B	109.7
S1—Cu1—I2^i^	97.98 (3)	C2—C3—C4	109.7 (3)
S1—Cu1—I2	117.03 (3)	H3A—C3—H3B	108.2
S1—Cu1—Cu1^i^	140.07 (3)	C4—C3—H3A	109.7
S1—Cu1—Cu2	159.65 (4)	C4—C3—H3B	109.7
S1—Cu1—Cu2^i^	135.38 (3)	C3—C4—H4A	109.0
I1^i^—Cu2—I1	118.71 (2)	C3—C4—H4B	109.0
I1^i^—Cu2—Cu1^i^	58.512 (18)	H4A—C4—H4B	107.8
I1^i^—Cu2—Cu1	109.64 (2)	C5—C4—C3	112.9 (3)
I1—Cu2—Cu1	57.350 (16)	C5—C4—H4A	109.0
I2—Cu2—I1^i^	115.15 (2)	C5—C4—H4B	109.0
I2—Cu2—I1	105.38 (2)	O1—C5—N1	117.9 (3)
I2—Cu2—Cu1^i^	62.509 (18)	O1—C5—C4	125.1 (3)
I2—Cu2—Cu1	58.432 (17)	N1—C5—C4	117.0 (3)
Cu1^i^—Cu2—I1	111.24 (2)	N2—C6—C7	178.5 (4)
Cu1^i^—Cu2—Cu1	61.59 (3)	C6—C7—H7A	109.5
Cu2^i^—Cu2—I1	61.16 (2)	C6—C7—H7B	109.5
Cu2^i^—Cu2—I1^i^	62.06 (2)	C6—C7—H7C	109.5
Cu2^i^—Cu2—I2	111.572 (12)	H7A—C7—H7B	109.5
Cu2^i^—Cu2—Cu1	60.528 (19)	H7A—C7—H7C	109.5
Cu2^i^—Cu2—Cu1^i^	63.50 (2)	H7B—C7—H7C	109.5
			
Cu1—S1—C1—N1	−11.4 (4)	C1—C2—C3—C4	−53.9 (4)
Cu1—S1—C1—C2	169.3 (3)	C2—C3—C4—C5	51.0 (5)
S1—C1—C2—C3	−152.4 (3)	C3—C4—C5—O1	156.9 (4)
N1—C1—C2—C3	28.3 (5)	C3—C4—C5—N1	−22.9 (5)
C1—N1—C5—O1	175.3 (4)	C5—N1—C1—S1	−177.2 (3)
C1—N1—C5—C4	−4.9 (6)	C5—N1—C1—C2	2.1 (6)

Symmetry code: (i) −*x*+1, *y*, −*z*+1/2.

### Bis(acetonitrile-κN)tetra-µ3-iodido-bis(6-sulfanylidenepiperidin-2-one-κS)-tetrahedro-tetracopper(I) (II) Hydrogen-bond geometry (Å, º)

**Table:**

*D*—H···*A*	*D*—H	H···*A*	*D*···*A*	*D*—H···*A*
N1—H1···I2	0.87 (2)	2.81 (2)	3.672 (3)	170 (4)

### catena-Poly[[(µ-6-sulfanylidenepiperidin-2-one-κ2O:S)copper(I)]-µ3-iodido] (III) Crystal data

**Table:**

[CuI(C_5_H_7_NOS)]	*D*_x_ = 2.612 Mg m^−^^3^
*M**_r_* = 319.62	Cu *K*α radiation, λ = 1.54178 Å
Orthorhombic, *P**b**c**n*	Cell parameters from 6676 reflections
*a* = 26.982 (11) Å	θ = 3.3–73.1°
*b* = 8.195 (4) Å	µ = 35.47 mm^−^^1^
*c* = 7.351 (3) Å	*T* = 100 K
*V* = 1625.4 (13) Å^3^	Plate, orange
*Z* = 8	0.08 × 0.07 × 0.03 mm
*F*(000) = 1200	

### catena-Poly[[(µ-6-sulfanylidenepiperidin-2-one-κ2O:S)copper(I)]-µ3-iodido] (III) Data collection

**Table:**

Bruker SMART APEXII area detector diffractometer	1631 independent reflections
Radiation source: sealed X-ray tube, Siemens, K FFCU 2K 90	1467 reflections with *I* > 2σ(*I*)
Equatorially mounted graphite monochromator	*R*_int_ = 0.051
Detector resolution: 7.9 pixels mm^-1^	θ_max_ = 73.6°, θ_min_ = 3.3°
0.60° ω and 0.6° φ scans	*h* = −32→33
Absorption correction: multi-scan SADABS; Bruker, 2016)	*k* = −10→9
*T*_min_ = 0.030, *T*_max_ = 0.144	*l* = −9→9
25274 measured reflections	

### catena-Poly[[(µ-6-sulfanylidenepiperidin-2-one-κ2O:S)copper(I)]-µ3-iodido] (III) Refinement

**Table:**

Refinement on *F*^2^	1 restraint
Least-squares matrix: full	Hydrogen site location: mixed
*R*[*F*^2^ > 2σ(*F*^2^)] = 0.032	H atoms treated by a mixture of independent and constrained refinement
*wR*(*F*^2^) = 0.082	*w* = 1/[σ^2^(*F*_o_^2^) + (0.055*P*)^2^ + 1.1889*P*] where *P* = (*F*_o_^2^ + 2*F*_c_^2^)/3
*S* = 1.07	(Δ/σ)_max_ = 0.001
1631 reflections	Δρ_max_ = 1.31 e Å^−^^3^
94 parameters	Δρ_min_ = −1.03 e Å^−^^3^

### catena-Poly[[(µ-6-sulfanylidenepiperidin-2-one-κ2O:S)copper(I)]-µ3-iodido] (III) Special details

**Table:**

Geometry. All esds (except the esd in the dihedral angle between two l.s. planes) are estimated using the full covariance matrix. The cell esds are taken into account individually in the estimation of esds in distances, angles and torsion angles; correlations between esds in cell parameters are only used when they are defined by crystal symmetry. An approximate (isotropic) treatment of cell esds is used for estimating esds involving l.s. planes.

### catena-Poly[[(µ-6-sulfanylidenepiperidin-2-one-κ2O:S)copper(I)]-µ3-iodido] (III) Fractional atomic coordinates and isotropic or equivalent isotropic displacement parameters (Å^2^)

**Table:**

	*x*	*y*	*z*	*U*_iso_*/*U*_eq_	
I1	0.23415 (2)	0.59898 (3)	0.50901 (3)	0.02390 (13)	
Cu1	0.28100 (2)	0.62683 (8)	0.82066 (9)	0.02635 (18)	
S1	0.36555 (4)	0.63113 (12)	0.83026 (14)	0.0266 (2)	
O1	0.50567 (13)	0.2890 (4)	0.9865 (4)	0.0346 (8)	
N1	0.43783 (13)	0.4297 (5)	0.9030 (5)	0.0274 (8)	
H1	0.4550 (18)	0.516 (4)	0.923 (7)	0.033*	
C1	0.38916 (15)	0.4443 (5)	0.8540 (6)	0.0253 (8)	
C2	0.35970 (15)	0.2932 (5)	0.8285 (6)	0.0269 (8)	
H2A	0.334849	0.311483	0.731519	0.032*	
H2B	0.341637	0.268491	0.942412	0.032*	
C3	0.39185 (16)	0.1470 (5)	0.7775 (7)	0.0303 (9)	
H3A	0.405740	0.162739	0.654072	0.036*	
H3B	0.371432	0.046611	0.776524	0.036*	
C4	0.43403 (17)	0.1289 (6)	0.9153 (7)	0.0333 (10)	
H4A	0.420142	0.095872	1.034455	0.040*	
H4B	0.456863	0.041794	0.874268	0.040*	
C5	0.46238 (17)	0.2851 (6)	0.9374 (6)	0.0299 (9)	

### catena-Poly[[(µ-6-sulfanylidenepiperidin-2-one-κ2O:S)copper(I)]-µ3-iodido] (III) Atomic displacement parameters (Å^2^)

**Table:**

	*U*^11^	*U*^22^	*U*^33^	*U*^12^	*U*^13^	*U*^23^
I1	0.02516 (19)	0.0217 (2)	0.02479 (19)	0.00135 (8)	0.00003 (9)	−0.00010 (9)
Cu1	0.0258 (3)	0.0243 (3)	0.0290 (4)	0.0002 (2)	−0.0002 (2)	0.0001 (3)
S1	0.0245 (5)	0.0238 (5)	0.0315 (5)	0.0009 (4)	−0.0001 (4)	−0.0002 (4)
O1	0.0273 (16)	0.0285 (16)	0.048 (2)	0.0009 (14)	−0.0039 (13)	−0.0007 (14)
N1	0.0253 (18)	0.0211 (17)	0.036 (2)	−0.0028 (13)	0.0010 (15)	−0.0012 (15)
C1	0.0257 (19)	0.025 (2)	0.0252 (19)	0.0017 (16)	0.0007 (16)	−0.0006 (17)
C2	0.0245 (19)	0.025 (2)	0.031 (2)	−0.0007 (16)	0.0000 (17)	−0.0015 (17)
C3	0.029 (2)	0.023 (2)	0.039 (2)	−0.0018 (16)	0.0003 (18)	−0.0042 (19)
C4	0.029 (2)	0.025 (2)	0.046 (3)	−0.0004 (18)	−0.001 (2)	0.001 (2)
C5	0.028 (2)	0.028 (2)	0.034 (2)	0.0032 (17)	0.0023 (17)	0.0001 (19)

### catena-Poly[[(µ-6-sulfanylidenepiperidin-2-one-κ2O:S)copper(I)]-µ3-iodido] (III) Geometric parameters (Å, º)

**Table:**

I1—Cu1	2.6264 (11)	C2—H2A	0.9900
I1—Cu1^i^	2.6709 (12)	C2—H2B	0.9900
I1—Cu1^ii^	2.6342 (10)	C2—C3	1.526 (6)
Cu1—S1	2.2827 (15)	C3—H3A	0.9900
S1—C1	1.668 (4)	C3—H3B	0.9900
O1—C5	1.223 (6)	C3—C4	1.531 (6)
N1—H1	0.86 (2)	C4—H4A	0.9900
N1—C1	1.367 (6)	C4—H4B	0.9900
N1—C5	1.381 (6)	C4—C5	1.500 (6)
C1—C2	1.483 (6)		
			
Cu1—I1—Cu1^ii^	106.77 (3)	H2A—C2—H2B	107.8
Cu1—I1—Cu1^i^	116.92 (2)	C3—C2—H2A	109.1
Cu1^ii^—I1—Cu1^i^	113.08 (4)	C3—C2—H2B	109.1
I1^iii^—Cu1—I1^iv^	104.19 (4)	C2—C3—H3A	109.7
I1—Cu1—I1^iv^	116.85 (3)	C2—C3—H3B	109.7
I1—Cu1—I1^iii^	99.58 (3)	C2—C3—C4	109.6 (4)
S1—Cu1—I1^iv^	97.12 (4)	H3A—C3—H3B	108.2
S1—Cu1—I1	120.62 (4)	C4—C3—H3A	109.7
S1—Cu1—I1^iii^	118.31 (4)	C4—C3—H3B	109.7
C1—S1—Cu1	111.76 (15)	C3—C4—H4A	109.3
C1—N1—H1	120 (4)	C3—C4—H4B	109.3
C1—N1—C5	125.7 (4)	H4A—C4—H4B	108.0
C5—N1—H1	115 (4)	C5—C4—C3	111.6 (4)
N1—C1—S1	118.4 (3)	C5—C4—H4A	109.3
N1—C1—C2	118.3 (4)	C5—C4—H4B	109.3
C2—C1—S1	123.3 (3)	O1—C5—N1	119.3 (4)
C1—C2—H2A	109.1	O1—C5—C4	122.8 (4)
C1—C2—H2B	109.1	N1—C5—C4	117.9 (4)
C1—C2—C3	112.5 (3)		
			
Cu1—S1—C1—N1	−165.4 (3)	C1—C2—C3—C4	52.5 (5)
Cu1—S1—C1—C2	13.3 (4)	C2—C3—C4—C5	−52.7 (5)
S1—C1—C2—C3	155.5 (3)	C3—C4—C5—O1	−154.2 (5)
N1—C1—C2—C3	−25.9 (5)	C3—C4—C5—N1	26.9 (6)
C1—N1—C5—O1	−177.4 (4)	C5—N1—C1—S1	176.5 (4)
C1—N1—C5—C4	1.5 (7)	C5—N1—C1—C2	−2.2 (7)

Symmetry codes: (i) −*x*+1/2, −*y*+3/2, *z*−1/2; (ii) *x*, −*y*+1, *z*−1/2; (iii) *x*, −*y*+1, *z*+1/2; (iv) −*x*+1/2, −*y*+3/2, *z*+1/2.

### catena-Poly[[(µ-6-sulfanylidenepiperidin-2-one-κ2O:S)copper(I)]-µ3-iodido] (III) Hydrogen-bond geometry (Å, º)

**Table:**

*D*—H···*A*	*D*—H	H···*A*	*D*···*A*	*D*—H···*A*
N1—H1···O1^v^	0.86 (2)	2.03 (2)	2.881 (5)	171 (5)

Symmetry code: (v) −*x*+1, −*y*+1, −*z*+2.

### Poly[[(piperidine-2,6-dithione-κS)copper(I)]-µ3-iodido] (IV) Crystal data

**Table:**

[Cu_2_I_2_(C_5_H_7_NS_2_)_2_]	*F*(000) = 1264
*M**_r_* = 671.35	*D*_x_ = 1.952 Mg m^−^^3^
Monoclinic, *P*2_1_/*c*	Cu *K*α radiation, λ = 1.54178 Å
*a* = 13.2866 (9) Å	Cell parameters from 9761 reflections
*b* = 11.6974 (13) Å	θ = 3.4–73.6°
*c* = 14.8089 (9) Å	µ = 26.87 mm^−^^1^
β = 96.998 (6)°	*T* = 100 K
*V* = 2284.4 (3) Å^3^	Block, red
*Z* = 4	0.22 × 0.13 × 0.09 mm

### Poly[[(piperidine-2,6-dithione-κS)copper(I)]-µ3-iodido] (IV) Data collection

**Table:**

Bruker SMART APEXII area detector diffractometer	4597 independent reflections
Radiation source: sealed X-ray tube, Siemens, K FFCU 2K 90	4236 reflections with *I* > 2σ(*I*)
Equatorially mounted graphite monochromator	*R*_int_ = 0.063
Detector resolution: 7.9 pixels mm^-1^	θ_max_ = 73.6°, θ_min_ = 3.4°
0.60° ω and 0.6° φ scans	*h* = −16→16
Absorption correction: multi-scan (SADABS; Bruker, 2016)	*k* = −14→14
*T*_min_ = 0.009, *T*_max_ = 0.094	*l* = −18→18
78133 measured reflections	

### Poly[[(piperidine-2,6-dithione-κS)copper(I)]-µ3-iodido] (IV) Refinement

**Table:**

Refinement on *F*^2^	Primary atom site location: dual
Least-squares matrix: full	Hydrogen site location: inferred from neighbouring sites
*R*[*F*^2^ > 2σ(*F*^2^)] = 0.048	H-atom parameters constrained
*wR*(*F*^2^) = 0.126	*w* = 1/[σ^2^(*F*_o_^2^) + (0.0963*P*)^2^ + 0.866*P*] where *P* = (*F*_o_^2^ + 2*F*_c_^2^)/3
*S* = 1.06	(Δ/σ)_max_ = 0.001
4597 reflections	Δρ_max_ = 1.58 e Å^−^^3^
181 parameters	Δρ_min_ = −0.48 e Å^−^^3^
0 restraints	

### Poly[[(piperidine-2,6-dithione-κS)copper(I)]-µ3-iodido] (IV) Special details

**Table:**

Geometry. All esds (except the esd in the dihedral angle between two l.s. planes) are estimated using the full covariance matrix. The cell esds are taken into account individually in the estimation of esds in distances, angles and torsion angles; correlations between esds in cell parameters are only used when they are defined by crystal symmetry. An approximate (isotropic) treatment of cell esds is used for estimating esds involving l.s. planes.

### Poly[[(piperidine-2,6-dithione-κS)copper(I)]-µ3-iodido] (IV) Fractional atomic coordinates and isotropic or equivalent isotropic displacement parameters (Å^2^)

**Table:**

	*x*	*y*	*z*	*U*_iso_*/*U*_eq_	
I2	0.55860 (2)	0.62760 (3)	0.40198 (2)	0.04041 (13)	
I1	−0.04785 (2)	0.16932 (3)	0.55075 (2)	0.04179 (13)	
Cu1	0.09352 (5)	0.01033 (6)	0.55762 (5)	0.03868 (18)	
Cu2	0.50067 (5)	0.41451 (7)	0.43516 (5)	0.04040 (19)	
S4	0.88076 (9)	0.04448 (10)	0.29165 (8)	0.0381 (3)	
S2	0.38476 (10)	0.38690 (11)	0.31034 (9)	0.0419 (3)	
S3	0.64474 (10)	0.30477 (12)	0.45389 (8)	0.0444 (3)	
S1	0.24194 (9)	0.09681 (11)	0.52729 (8)	0.0410 (3)	
N1	0.2946 (3)	0.2359 (4)	0.4050 (3)	0.0400 (9)	
H1	0.341595	0.255812	0.449306	0.048*	
N2	0.7560 (3)	0.1895 (4)	0.3534 (3)	0.0393 (9)	
H2	0.784079	0.164625	0.406656	0.047*	
C6	0.6808 (4)	0.2704 (4)	0.3544 (3)	0.0392 (10)	
C1	0.2258 (4)	0.1562 (5)	0.4253 (3)	0.0399 (10)	
C10	0.7913 (4)	0.1441 (4)	0.2793 (4)	0.0373 (10)	
C5	0.2980 (4)	0.2881 (4)	0.3228 (4)	0.0400 (10)	
C2	0.1396 (4)	0.1307 (5)	0.3541 (4)	0.0451 (12)	
H2A	0.077033	0.121868	0.383553	0.054*	
H2B	0.152702	0.056988	0.324839	0.054*	
C9	0.7453 (4)	0.1855 (5)	0.1882 (3)	0.0414 (11)	
H9A	0.687396	0.135704	0.165575	0.050*	
H9B	0.796127	0.180538	0.144760	0.050*	
C7	0.6364 (5)	0.3190 (5)	0.2646 (4)	0.0473 (12)	
H7A	0.620041	0.400624	0.272580	0.057*	
H7B	0.572524	0.278409	0.243395	0.057*	
C4	0.2216 (4)	0.2518 (5)	0.2464 (4)	0.0449 (11)	
H4A	0.247023	0.184112	0.216075	0.054*	
H4B	0.211015	0.314198	0.200990	0.054*	
C8	0.7087 (5)	0.3083 (5)	0.1928 (4)	0.0480 (12)	
H8A	0.673595	0.331533	0.132807	0.058*	
H8B	0.767460	0.359753	0.208203	0.058*	
C3	0.1218 (4)	0.2229 (5)	0.2805 (4)	0.0474 (12)	
H3A	0.072927	0.194853	0.229493	0.057*	
H3B	0.092969	0.292195	0.305907	0.057*	

### Poly[[(piperidine-2,6-dithione-κS)copper(I)]-µ3-iodido] (IV) Atomic displacement parameters (Å^2^)

**Table:**

	*U*^11^	*U*^22^	*U*^33^	*U*^12^	*U*^13^	*U*^23^
I2	0.0431 (2)	0.0411 (2)	0.0377 (2)	−0.00315 (12)	0.00764 (14)	0.00019 (12)
I1	0.0442 (2)	0.0404 (2)	0.0395 (2)	0.00544 (12)	0.00003 (14)	−0.00445 (12)
Cu1	0.0398 (4)	0.0393 (4)	0.0368 (4)	0.0010 (3)	0.0043 (3)	0.0020 (3)
Cu2	0.0397 (4)	0.0416 (4)	0.0404 (4)	0.0001 (3)	0.0066 (3)	−0.0006 (3)
S4	0.0389 (6)	0.0400 (6)	0.0356 (5)	0.0026 (4)	0.0055 (4)	0.0004 (5)
S2	0.0425 (6)	0.0427 (6)	0.0406 (6)	−0.0043 (5)	0.0054 (5)	0.0042 (5)
S3	0.0462 (7)	0.0530 (7)	0.0346 (6)	0.0106 (5)	0.0069 (5)	0.0011 (5)
S1	0.0416 (6)	0.0460 (7)	0.0352 (6)	−0.0036 (5)	0.0034 (5)	0.0044 (5)
N1	0.039 (2)	0.044 (2)	0.0367 (19)	−0.0026 (17)	0.0041 (16)	0.0006 (18)
N2	0.039 (2)	0.044 (2)	0.0346 (19)	0.0019 (17)	0.0018 (16)	0.0039 (17)
C6	0.041 (2)	0.038 (2)	0.038 (2)	0.0000 (19)	0.0046 (19)	0.003 (2)
C1	0.041 (3)	0.044 (3)	0.035 (2)	0.000 (2)	0.004 (2)	0.001 (2)
C10	0.036 (2)	0.037 (2)	0.039 (2)	−0.0020 (18)	0.0048 (19)	0.001 (2)
C5	0.038 (2)	0.042 (3)	0.040 (2)	0.001 (2)	0.0050 (19)	−0.001 (2)
C2	0.047 (3)	0.051 (3)	0.037 (3)	−0.008 (2)	0.003 (2)	0.003 (2)
C9	0.046 (3)	0.044 (3)	0.034 (2)	0.005 (2)	0.006 (2)	−0.001 (2)
C7	0.055 (3)	0.049 (3)	0.037 (3)	0.013 (2)	0.004 (2)	0.002 (2)
C4	0.049 (3)	0.045 (3)	0.040 (3)	−0.005 (2)	0.004 (2)	0.006 (2)
C8	0.062 (3)	0.047 (3)	0.035 (2)	0.012 (3)	0.010 (2)	0.006 (2)
C3	0.042 (3)	0.057 (3)	0.042 (3)	−0.004 (2)	0.001 (2)	0.002 (2)

### Poly[[(piperidine-2,6-dithione-κS)copper(I)]-µ3-iodido] (IV) Geometric parameters (Å, º)

**Table:**

I1—Cu1	2.6365 (8)	C1—C2	1.490 (7)
I1—Cu1^i^	2.6687 (8)	C10—C9	1.491 (7)
I2—Cu2	2.6719 (8)	C5—C4	1.487 (7)
I2—Cu2^ii^	2.6724 (8)	C2—H2A	0.9900
Cu1—S4^iii^	2.3075 (13)	C2—H2B	0.9900
Cu1—S1	2.3086 (14)	C2—C3	1.531 (8)
Cu2—S2	2.2802 (15)	C9—H9A	0.9900
Cu2—S3	2.2933 (15)	C9—H9B	0.9900
S4—C10	1.659 (5)	C9—C8	1.521 (7)
S2—C5	1.659 (5)	C7—H7A	0.9900
S3—C6	1.654 (5)	C7—H7B	0.9900
S1—C1	1.652 (5)	C7—C8	1.522 (8)
N1—H1	0.8800	C4—H4A	0.9900
N1—C1	1.365 (7)	C4—H4B	0.9900
N1—C5	1.367 (7)	C4—C3	1.514 (8)
N2—H2	0.8800	C8—H8A	0.9900
N2—C6	1.378 (7)	C8—H8B	0.9900
N2—C10	1.354 (7)	C3—H3A	0.9900
C6—C7	1.498 (7)	C3—H3B	0.9900
			
Cu2—I2—Cu2^ii^	62.54 (3)	C1—C2—H2A	108.8
Cu1—I1—Cu1^i^	64.94 (3)	C1—C2—H2B	108.8
I1—Cu1—I1^i^	115.06 (3)	C1—C2—C3	113.7 (5)
S4^iii^—Cu1—I1	104.72 (4)	H2A—C2—H2B	107.7
S4^iii^—Cu1—I1^i^	111.04 (4)	C3—C2—H2A	108.8
S4^iii^—Cu1—S1	106.30 (5)	C3—C2—H2B	108.8
S1—Cu1—I1^i^	111.41 (4)	C10—C9—H9A	109.4
S1—Cu1—I1	107.75 (4)	C10—C9—H9B	109.4
I2—Cu2—I2^ii^	117.46 (3)	C10—C9—C8	111.3 (4)
S2—Cu2—I2^ii^	117.47 (4)	H9A—C9—H9B	108.0
S2—Cu2—I2	99.46 (4)	C8—C9—H9A	109.4
S2—Cu2—S3	119.35 (6)	C8—C9—H9B	109.4
S3—Cu2—I2^ii^	96.95 (4)	C6—C7—H7A	109.2
S3—Cu2—I2	106.83 (5)	C6—C7—H7B	109.2
C10—S4—Cu1^iii^	108.78 (18)	C6—C7—C8	112.1 (5)
C5—S2—Cu2	114.75 (19)	H7A—C7—H7B	107.9
C6—S3—Cu2	110.88 (19)	C8—C7—H7A	109.2
C1—S1—Cu1	110.1 (2)	C8—C7—H7B	109.2
C1—N1—H1	116.7	C5—C4—H4A	109.5
C1—N1—C5	126.6 (5)	C5—C4—H4B	109.5
C5—N1—H1	116.7	C5—C4—C3	110.7 (5)
C6—N2—H2	116.5	H4A—C4—H4B	108.1
C10—N2—H2	116.5	C3—C4—H4A	109.5
C10—N2—C6	127.0 (4)	C3—C4—H4B	109.5
N2—C6—S3	117.7 (4)	C9—C8—C7	109.9 (5)
N2—C6—C7	117.3 (4)	C9—C8—H8A	109.7
C7—C6—S3	125.0 (4)	C9—C8—H8B	109.7
N1—C1—S1	118.2 (4)	C7—C8—H8A	109.7
N1—C1—C2	117.3 (5)	C7—C8—H8B	109.7
C2—C1—S1	124.5 (4)	H8A—C8—H8B	108.2
N2—C10—S4	120.0 (4)	C2—C3—H3A	109.8
N2—C10—C9	117.5 (4)	C2—C3—H3B	109.8
C9—C10—S4	122.5 (4)	C4—C3—C2	109.2 (5)
N1—C5—S2	120.6 (4)	C4—C3—H3A	109.8
N1—C5—C4	117.2 (4)	C4—C3—H3B	109.8
C4—C5—S2	122.2 (4)	H3A—C3—H3B	108.3
			
Cu1^iii^—S4—C10—N2	8.2 (5)	N2—C6—C7—C8	−23.4 (7)
Cu1^iii^—S4—C10—C9	−170.5 (4)	N2—C10—C9—C8	29.6 (7)
Cu1—S1—C1—N1	162.9 (4)	C6—N2—C10—S4	−178.4 (4)
Cu1—S1—C1—C2	−15.7 (5)	C6—N2—C10—C9	0.4 (8)
Cu2—S2—C5—N1	4.8 (5)	C6—C7—C8—C9	51.7 (7)
Cu2—S2—C5—C4	−174.9 (4)	C1—N1—C5—S2	177.9 (4)
Cu2—S3—C6—N2	−169.5 (3)	C1—N1—C5—C4	−2.4 (8)
Cu2—S3—C6—C7	9.3 (5)	C1—C2—C3—C4	48.8 (7)
S4—C10—C9—C8	−151.7 (4)	C10—N2—C6—S3	175.3 (4)
S2—C5—C4—C3	−145.6 (4)	C10—N2—C6—C7	−3.7 (8)
S3—C6—C7—C8	157.7 (4)	C10—C9—C8—C7	−54.8 (6)
S1—C1—C2—C3	160.4 (4)	C5—N1—C1—S1	175.1 (4)
N1—C1—C2—C3	−18.2 (7)	C5—N1—C1—C2	−6.3 (8)
N1—C5—C4—C3	34.7 (7)	C5—C4—C3—C2	−56.3 (6)

Symmetry codes: (i) −*x*, −*y*, −*z*+1; (ii) −*x*+1, −*y*+1, −*z*+1; (iii) −*x*+1, −*y*, −*z*+1.

### Poly[[(piperidine-2,6-dithione-κS)copper(I)]-µ3-iodido] (IV) Hydrogen-bond geometry (Å, º)

**Table:**

*D*—H···*A*	*D*—H	H···*A*	*D*···*A*	*D*—H···*A*
N1—H1···I2^ii^	0.88	2.79	3.628 (4)	161
N2—H2···I1^iv^	0.88	2.90	3.679 (4)	149

Symmetry codes: (ii) −*x*+1, −*y*+1, −*z*+1; (iv) *x*+1, *y*, *z*.

### Poly[[(µ-isoindoline-1,3-dithione-κ2S:S)copper(I)]-µ3-iodido] (V) Crystal data

**Table:**

[CuI(C_8_H_5_NS_2_)]	*F*(000) = 696
*M**_r_* = 369.69	*D*_x_ = 2.492 Mg m^−^^3^
Monoclinic, *C**c*	Mo *K*α radiation, λ = 0.71073 Å
*a* = 15.174 (5) Å	Cell parameters from 4507 reflections
*b* = 4.1188 (16) Å	θ = 2.6–31.9°
*c* = 15.785 (6) Å	µ = 5.72 mm^−^^1^
β = 92.98 (2)°	*T* = 100 K
*V* = 985.2 (6) Å^3^	Block, black
*Z* = 4	0.30 × 0.02 × 0.01 mm

### Poly[[(µ-isoindoline-1,3-dithione-κ2S:S)copper(I)]-µ3-iodido] (V) Data collection

**Table:**

Bruker APEXII Quazar diffractometer	3603 independent reflections
Radiation source: microfocus sealed X-ray tube, Incoatec Iµs	3226 reflections with *I* > 2σ(*I*)
Mirror optics monochromator	*R*_int_ = 0.041
Detector resolution: 7.9 pixels mm^-1^	θ_max_ = 33.2°, θ_min_ = 2.6°
0.5° ω and 0.5° φ scans	*h* = −22→22
Absorption correction: multi-scan (SADABS; Bruker, 2016)	*k* = −6→6
*T*_min_ = 0.322, *T*_max_ = 0.404	*l* = −23→23
11871 measured reflections	

### Poly[[(µ-isoindoline-1,3-dithione-κ2S:S)copper(I)]-µ3-iodido] (V) Refinement

**Table:**

Refinement on *F*^2^	Hydrogen site location: inferred from neighbouring sites
Least-squares matrix: full	H-atom parameters constrained
*R*[*F*^2^ > 2σ(*F*^2^)] = 0.036	*w* = 1/[σ^2^(*F*_o_^2^) + (0.0417*P*)^2^ + 7.0163*P*] where *P* = (*F*_o_^2^ + 2*F*_c_^2^)/3
*wR*(*F*^2^) = 0.089	(Δ/σ)_max_ < 0.001
*S* = 1.06	Δρ_max_ = 3.17 e Å^−^^3^
3603 reflections	Δρ_min_ = −1.13 e Å^−^^3^
118 parameters	Absolute structure: Flack *x* determined using 1438 quotients [(*I*^+^)-(*I*^-^)]/[(*I*^+^)+(*I*^-^)] (Parsons *et al.*, 2013)
2 restraints	Absolute structure parameter: 0.034 (12)
Primary atom site location: dual	

### Poly[[(µ-isoindoline-1,3-dithione-κ2S:S)copper(I)]-µ3-iodido] (V) Special details

**Table:**

Geometry. All esds (except the esd in the dihedral angle between two l.s. planes) are estimated using the full covariance matrix. The cell esds are taken into account individually in the estimation of esds in distances, angles and torsion angles; correlations between esds in cell parameters are only used when they are defined by crystal symmetry. An approximate (isotropic) treatment of cell esds is used for estimating esds involving l.s. planes.

### Poly[[(µ-isoindoline-1,3-dithione-κ2S:S)copper(I)]-µ3-iodido] (V) Fractional atomic coordinates and isotropic or equivalent isotropic displacement parameters (Å^2^)

**Table:**

	*x*	*y*	*z*	*U*_iso_*/*U*_eq_	
I1	0.68957 (2)	0.10931 (10)	0.74974 (2)	0.01374 (11)	
Cu1	0.59491 (6)	0.5992 (2)	0.69164 (6)	0.0146 (2)	
S1	0.60747 (14)	0.6607 (5)	0.54996 (12)	0.0139 (3)	
S2	0.46550 (14)	0.2757 (5)	0.25028 (12)	0.0146 (3)	
N1	0.5450 (5)	0.4774 (16)	0.3969 (4)	0.0133 (12)	
H1	0.587624	0.579000	0.371951	0.016*	
C1	0.5382 (5)	0.4700 (18)	0.4850 (5)	0.0122 (13)	
C2	0.4596 (5)	0.2691 (18)	0.4975 (5)	0.0124 (14)	
C3	0.4197 (5)	0.1812 (19)	0.5712 (5)	0.0147 (14)	
H3	0.442699	0.252848	0.625113	0.018*	
C4	0.3448 (5)	−0.015 (2)	0.5639 (5)	0.0148 (14)	
H4	0.316667	−0.082004	0.613403	0.018*	
C5	0.3106 (5)	−0.115 (2)	0.4828 (5)	0.0165 (15)	
H5	0.259718	−0.249734	0.478913	0.020*	
C6	0.3493 (5)	−0.0227 (18)	0.4093 (5)	0.0128 (13)	
H6	0.325668	−0.090075	0.355269	0.015*	
C7	0.4244 (5)	0.1733 (18)	0.4172 (5)	0.0125 (13)	
C8	0.4795 (5)	0.3123 (19)	0.3538 (5)	0.0118 (13)	

### Poly[[(µ-isoindoline-1,3-dithione-κ2S:S)copper(I)]-µ3-iodido] (V) Atomic displacement parameters (Å^2^)

**Table:**

	*U*^11^	*U*^22^	*U*^33^	*U*^12^	*U*^13^	*U*^23^
I1	0.0131 (2)	0.01109 (17)	0.0169 (2)	−0.0009 (2)	−0.00081 (15)	−0.0008 (2)
Cu1	0.0155 (5)	0.0162 (5)	0.0122 (5)	−0.0008 (4)	0.0004 (4)	−0.0010 (4)
S1	0.0141 (9)	0.0150 (8)	0.0124 (8)	0.0001 (6)	0.0003 (7)	−0.0011 (6)
S2	0.0132 (8)	0.0185 (9)	0.0120 (8)	−0.0011 (7)	−0.0002 (7)	−0.0017 (7)
N1	0.014 (3)	0.014 (3)	0.012 (3)	0.001 (2)	0.001 (2)	0.001 (2)
C1	0.012 (3)	0.012 (3)	0.013 (3)	0.003 (2)	0.000 (3)	0.002 (3)
C2	0.012 (4)	0.009 (3)	0.017 (3)	0.006 (3)	0.002 (3)	0.000 (2)
C3	0.014 (3)	0.013 (3)	0.017 (3)	0.002 (3)	0.002 (3)	0.001 (3)
C4	0.010 (3)	0.018 (3)	0.017 (4)	0.001 (3)	0.003 (3)	0.002 (3)
C5	0.012 (3)	0.018 (4)	0.019 (4)	−0.002 (3)	−0.003 (3)	0.001 (3)
C6	0.011 (3)	0.011 (3)	0.017 (3)	0.000 (2)	−0.001 (3)	0.001 (3)
C7	0.015 (3)	0.009 (3)	0.014 (3)	0.002 (2)	0.002 (3)	0.001 (2)
C8	0.011 (3)	0.013 (3)	0.012 (3)	0.003 (2)	0.002 (3)	0.000 (2)

### Poly[[(µ-isoindoline-1,3-dithione-κ2S:S)copper(I)]-µ3-iodido] (V) Geometric parameters (Å, º)

**Table:**

I1—Cu1	2.6152 (13)	C2—C7	1.405 (11)
I1—Cu1^i^	2.6798 (13)	C3—H3	0.9500
Cu1—S1	2.269 (2)	C3—C4	1.394 (12)
Cu1—S2^ii^	2.273 (2)	C4—H4	0.9500
S1—C1	1.632 (8)	C4—C5	1.417 (12)
S2—C8	1.645 (7)	C5—H5	0.9500
N1—H1	0.8800	C5—C6	1.380 (11)
N1—C1	1.400 (10)	C6—H6	0.9500
N1—C8	1.357 (10)	C6—C7	1.396 (11)
C1—C2	1.473 (11)	C7—C8	1.455 (11)
C2—C3	1.388 (11)		
			
Cu1—I1—Cu1^i^	102.12 (4)	C2—C3—H3	120.9
I1—Cu1—I1^iii^	102.12 (4)	C2—C3—C4	118.1 (8)
S1—Cu1—I1^iii^	100.30 (6)	C4—C3—H3	120.9
S1—Cu1—I1	111.05 (6)	C3—C4—H4	120.0
S1—Cu1—S2^ii^	119.65 (8)	C3—C4—C5	120.1 (8)
S2^ii^—Cu1—I1^iii^	98.16 (7)	C5—C4—H4	120.0
S2^ii^—Cu1—I1	120.16 (7)	C4—C5—H5	119.1
C1—S1—Cu1	118.8 (3)	C6—C5—C4	121.8 (8)
C8—S2—Cu1^iv^	108.3 (3)	C6—C5—H5	119.1
C1—N1—H1	123.3	C5—C6—H6	121.1
C8—N1—H1	123.3	C5—C6—C7	117.7 (7)
C8—N1—C1	113.3 (7)	C7—C6—H6	121.1
N1—C1—S1	122.3 (6)	C2—C7—C8	107.7 (7)
N1—C1—C2	104.3 (7)	C6—C7—C2	120.9 (7)
C2—C1—S1	133.3 (6)	C6—C7—C8	131.4 (7)
C3—C2—C1	130.6 (7)	N1—C8—S2	126.7 (6)
C3—C2—C7	121.4 (7)	N1—C8—C7	106.6 (6)
C7—C2—C1	108.0 (7)	C7—C8—S2	126.7 (6)
			
Cu1—S1—C1—N1	−175.6 (5)	C2—C7—C8—S2	−179.1 (6)
Cu1—S1—C1—C2	5.3 (9)	C2—C7—C8—N1	1.0 (8)
Cu1^iv^—S2—C8—N1	18.3 (8)	C3—C2—C7—C6	−2.0 (11)
Cu1^iv^—S2—C8—C7	−161.5 (6)	C3—C2—C7—C8	178.2 (7)
S1—C1—C2—C3	0.4 (13)	C3—C4—C5—C6	−0.2 (12)
S1—C1—C2—C7	178.6 (6)	C4—C5—C6—C7	0.4 (12)
N1—C1—C2—C3	−178.9 (8)	C5—C6—C7—C2	0.7 (11)
N1—C1—C2—C7	−0.6 (8)	C5—C6—C7—C8	−179.6 (8)
C1—N1—C8—S2	178.7 (6)	C6—C7—C8—S2	1.1 (12)
C1—N1—C8—C7	−1.5 (9)	C6—C7—C8—N1	−178.7 (8)
C1—C2—C3—C4	−179.8 (8)	C7—C2—C3—C4	2.1 (11)
C1—C2—C7—C6	179.5 (7)	C8—N1—C1—S1	−178.0 (6)
C1—C2—C7—C8	−0.2 (8)	C8—N1—C1—C2	1.3 (8)
C2—C3—C4—C5	−1.0 (12)		

Symmetry codes: (i) *x*, *y*−1, *z*; (ii) *x*, −*y*+1, *z*+1/2; (iii) *x*, *y*+1, *z*; (iv) *x*, −*y*+1, *z*−1/2.

### Poly[[(µ-isoindoline-1,3-dithione-κ2S:S)copper(I)]-µ3-iodido] (V) Hydrogen-bond geometry (Å, º)

**Table:**

*D*—H···*A*	*D*—H	H···*A*	*D*···*A*	*D*—H···*A*
N1—H1···I1^iv^	0.88	2.84	3.692 (7)	163

Symmetry code: (iv) *x*, −*y*+1, *z*−1/2.
